# The weaker-binding Fc γ receptor IIIa F158 allotype retains sensitivity to N-glycan composition and exhibits a destabilized antibody-binding interface

**DOI:** 10.1016/j.jbc.2022.102329

**Published:** 2022-07-31

**Authors:** Paul G. Kremer, Adam W. Barb

**Affiliations:** 1 Department of Biochemistry and Molecular Biology, University of Georgia, Athens, Georgia, USA; 2 Department of Chemistry, University of Georgia, Athens, Georgia, USA; 3 Complex Carbohydrate Research Center, University of Georgia, Athens, Georgia, USA

**Keywords:** NMR spectroscopy, surface plasmon resonance, glycobiology, glycoprotein, adaptive immune system, Fc, crystallizable fragment, FcγR, Fc γ receptor, IgG, immunoglobulin G, NK, natural killer, TEV, tobacco etch virus

## Abstract

Antibodies engage Fc γ receptors (FcγRs) to elicit healing cellular immune responses following binding to a target antigen. Fc γ receptor IIIa/CD16a triggers natural killer cells to destroy target tissues with cytotoxic proteins and enhances phagocytosis mediated by macrophages. Multiple variables affect CD16a antibody-binding strength and the resulting immune response, including a genetic polymorphism. The predominant CD16a F158 allotype binds antibodies with less affinity than the less common V158 allotype. This polymorphism likewise affects cellular immune responses and clinical efficacy of antibodies relying on CD16a engagement, though it remains unclear how V/F158 affects CD16a structure. Another relevant variable shown to affect affinity is composition of the CD16a asparagine-linked (N)-glycans. It is currently not known how N-glycan composition affects CD16a F158 affinity. Here, we determined N-glycan composition affects the V158 and F158 allotypes similarly, and N-glycan composition does not explain differences in V158 and F158 binding affinity. Our analysis of binding kinetics indicated the N162 glycan slows the binding event, and shortening the N-glycans or removing the N162 glycan increased the speed of binding. F158 displayed a slower binding rate than V158. Surprisingly, we found N-glycan composition had a smaller effect on the dissociation rate. We also identified conformational heterogeneity of CD16a F158 backbone amide and N162 glycan resonances using NMR spectroscopy. Residues exhibiting chemical shift perturbations between V158 and F158 mapped to the antibody-binding interface. These data support a model for CD16a F158 with increased interface conformational heterogeneity, reducing the population of binding-competent forms available and decreasing affinity.

Fc γ receptor (FcγR) IIIa/CD16a binds to the crystallizable fragment (Fc) of immunoglobulin G (IgG) antibodies (Fig. 1). CD16a triggers cell activation upon encountering antibodies clustered on a surface, which may include a damaged tissue, foreign pathogen, or particle. Possible cell-mediated immune responses are phagocytic and cytotoxic, leading to destruction of the antibody-coated target (1, 2). The strength of the cellular response depends on the antibody-binding affinity of CD16a.

**Figure 1:**
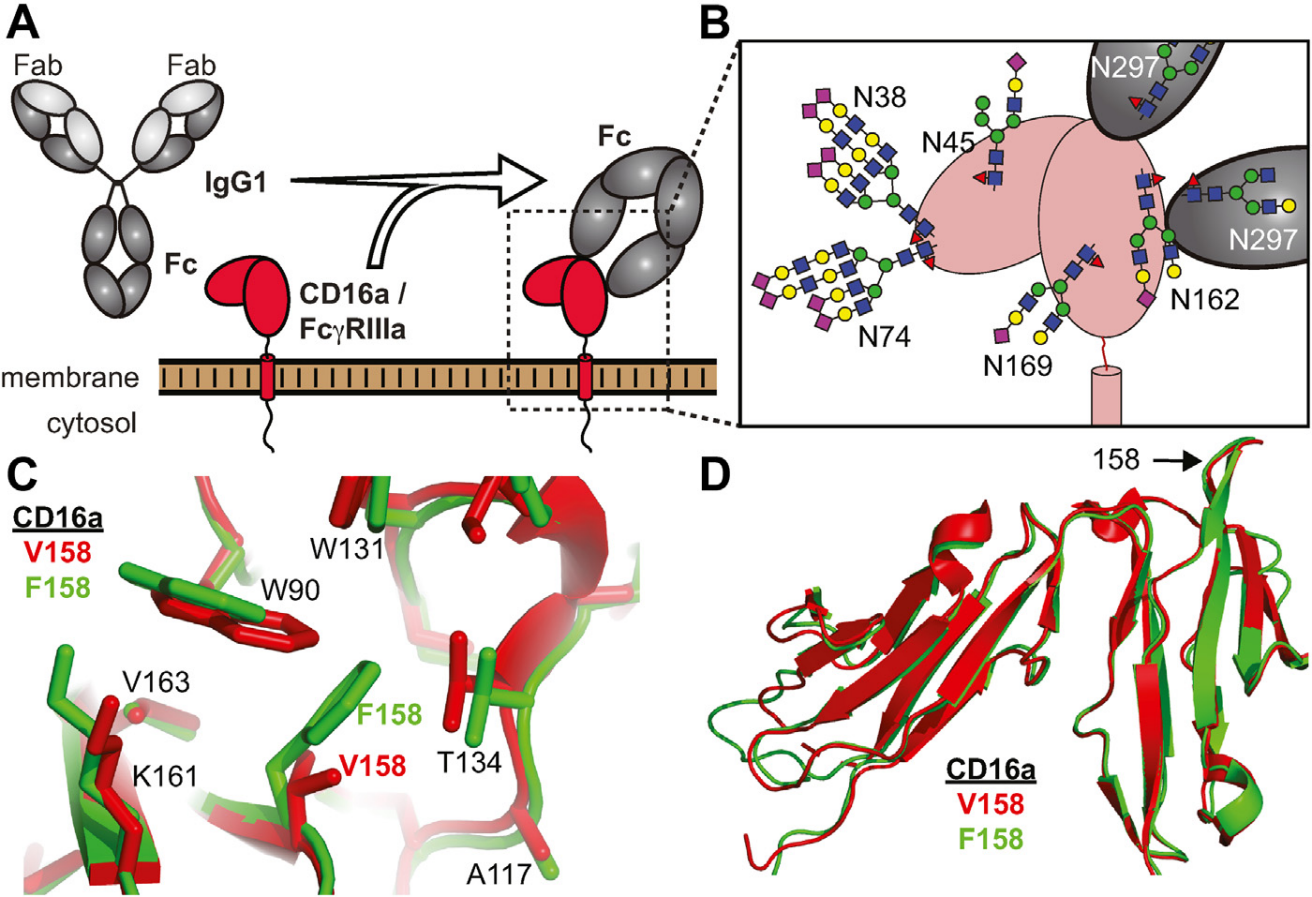
**Two common receptor allotypes in the human genome differ by a single amino acid substitution at position 158 in the extracellular antibody-binding domain.***A*, CD16a/Fc γ receptor (FcγR) IIIa binds the crystallizable fragment (Fc) of immunoglobulin G1 (IgG1). *B*, CD16a is heavily N-glycosylated with five N-glycans. The predominant glycoforms identified on primary human natural killer cells are shown as *cartoons* and scaled to the appropriate size. Fc is likewise glycosylated at N297. An overlay of the CD16a V158 structure (*red*, pdb 5vu0) with an AlphaFold model of CD16a F158 (*green*) shows residues surrounding the 158 position (*C*) and the global structural similarity (*D*).

Two major CD16a forms in humans result from a genetic polymorphism affecting the amino acid residue at position 158 on the extracellular antibody-binding domain (3). The allele frequency varies by population but several studies collectively suggest the V158-encoding allele is present at 30 to 39% frequency, with the remainder encoding F158 (3, 4, 5). In binding affinity assays, the CD16a F158 allotype binds IgG1 4 to 5 fold weaker than V158 (6, 7).

Natural Killer (NK) cells function in the innate immune system but respond to antibody-coated targets through CD16a as the primary FcγR. NK cells expressing F158 showed less cytotoxic activity following antibody treatment than V158 (8). Similarly, Binyamin *et al.* (9) showed increased antibody-dependent cell-mediated cytotoxicity for V158 with NK92 cells. Data from primary human NK cells also supports V158 promoting antibody-dependent cell-mediated cytotoxicity compared to F158 (7). Clinical studies link the F158 allotype to increased susceptibility to a wide assortment of diseases (10, 11, 12, 13, 14). Furthermore, V158 patients experienced superior responses to clinical antibody treatments (15, 16, 17). Though these correlations are known, it is not known how a single amino acid substitution in this receptor affects the ligand-binding affinity.

In addition to the CD16a residue at 158, glycosylation was recently shown to affect antibody-binding affinity. CD16a is a transmembrane receptor modified with five asparagine(N)-linked carbohydrates (glycans). N-glycans on secreted proteins exhibit a high degree of compositional heterogeneity due to the template-independent glycan processing in the ER and Golgi (18). CD16a exhibits a high degree of heterogeneity. Among the five N-glycosylation sites, N162 appears the most heterogeneous on CD16a isolated from primary human cells (19, 20). Furthermore, CD16a V158 N162 glycan heterogeneity impacts Fc-binding affinity. Minimally processed oligomannose glycans, present on CD16a isolated from human donors, provide greater affinity than highly processed complex-type glycans on CD16a (21, 22). Hayes *et al.* (23) also found binding differences related to CD16a glycosylation and the 158 allotype. It is not currently known how the N162 N-glycan composition affects the antibody-binding affinity of the CD16a F158 allotype.

We investigated the structural and functional consequences of a phenylalanine residue at CD16a position 158 relative to a valine. We first characterized the impact of N-glycans on antibody-binding affinity by CD16a F158. Next, we identified backbone and N-glycan atoms that were differentially affected by V158 and F158 using NMR spectroscopy. These data provide insight into the unique structural and functional characteristics of the predominant CD16a F158 allotype with atomic-level resolution.

## Results

### Structural similarity of V158 and F158

Multiple examples of the CD16a V158 structure from X-ray crystallography are reported and are highly similar (24, 25, 26). As of this writing, no models of the F158 variant determined by X-ray crystallography or cryo-EM are reported despite the single amino acid difference and greater prevalence in the human population. AlphaFold, however, provided a computational model for comparison (27). As expected, the structures of the extracellular antibody-binding domains are highly similar (Fig. 1, *C* and *D*). Subtle differences in the packing around position 158 are notable, stemming from steric contacts that prevent the F158 sidechain from occupying the same position as V158. This comparison does not clearly indicate why F158 binds weaker than V158. However, relatively small changes in binding affinity, like those noted for F158 and V158, may not result from large structural rearrangements and may be instead due to differences in conformational sampling or glycosylation that are not always identifiable in models from X-ray crystallography or AlphaFold.

### The effect of N-glycan composition on CD16a F158 affinity

We examined whether glycosylation explained the reported differences in affinity. Our laboratory previously determined that the composition of the N162 glycan affects CD16a V158 antibody-binding affinity (22). Thus, it is possible that F158 affects N-glycan processing, weakening affinity. WT HEK293F cells expressed both CD16a V158 and F158 and are capable of performing extensive N-glycan remodeling reactions and generating complex-type N-glycoforms. SDS-PAGE showed substantial processing as both proteins migrated much higher than expected based on the predicted molecular weight of the unmodified polypeptide (50.5 kDa; Fig. S1). Mass spectrometry analysis demonstrated that both allotypes contain similar N162-glycans, predominantly complex-type, core fucosylated glycoforms (Fig. S2 and Table S1). A small amount of oligomannose (6%) appeared in the V158 allotype. Furthermore, a recent analysis demonstrated a high degree of N-glycan occupancy for both allotypes (28).

**Figure 2:**
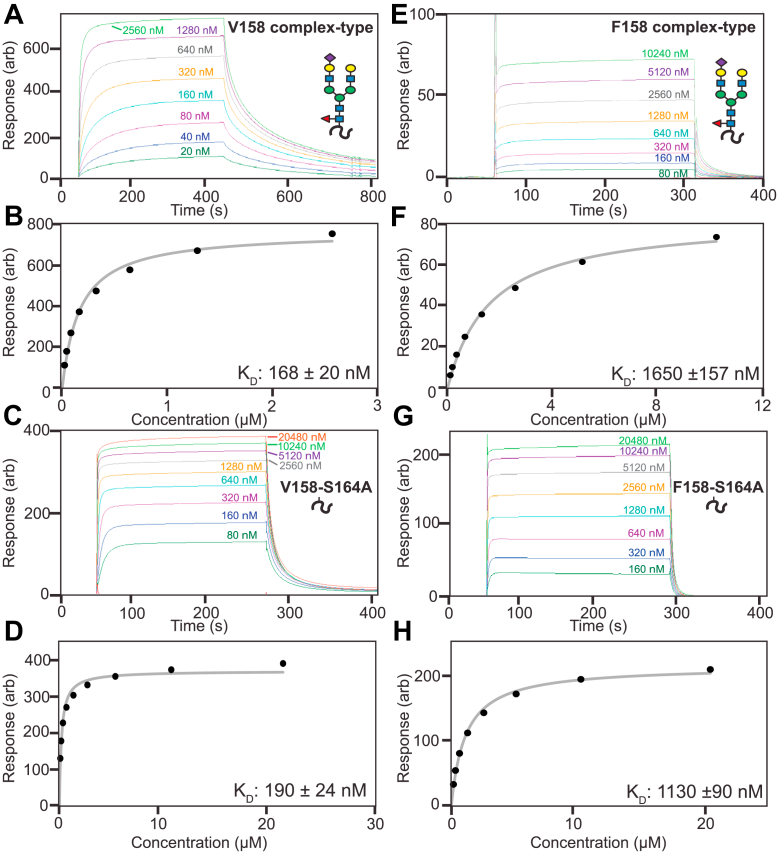
**Representative surface plasmon resonance (SPR) sensorgrams of CD16a allotypes.** Glycoforms are complex-types (*A* and *E*) with two variants missing the N162 glycan (*C* and *G*). Fits of the dissociation constants and associated errors from equilibrium binding measurements are also shown (*B*, *D*, *F*, and *H*). *Cartoons* show the expected N-glycan at the N162 site. The S164A mutation disrupts the N162 N-glycan sequon and thus lacks an N-glycan at that site.

**Figure 3:**
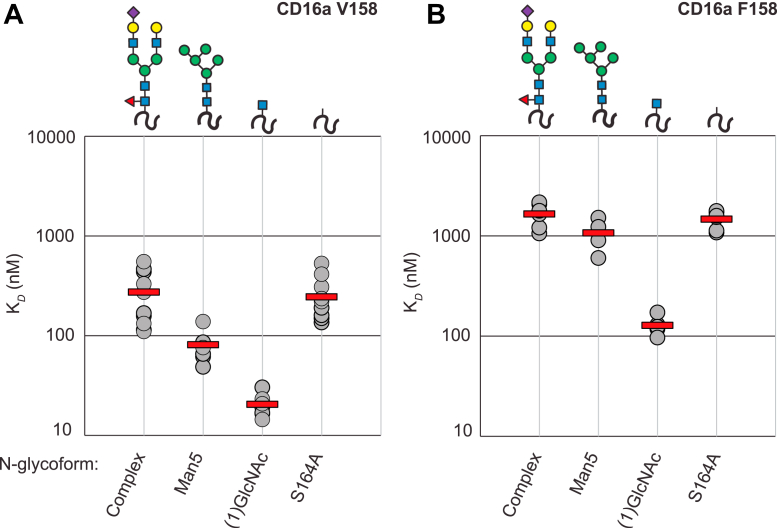
**Affinity of each CD16a allotype glycoform.** Each mean (*horizontal red bar*) represents a minimum of five individual measurements (*gray circles*). Glycan representations reflect the modification at the N162 site. *A*) V158, *B*) F158.

The CD16a F158 allotype bound IgG1 Fc with a six-fold reduction in affinity compared to V158 (Figs. 2 and 3 and Table 1), consistent with previous reports (6, 21, 29). We next expressed both CD16a allotypes in a Gnt1-cell line that expresses glycoproteins with predominantly oligomannose (Man5GlcNAc2) N-glycans (30). The mobility of these proteins in SDS-PAGE increased from the presence of smaller N-glycans (Fig. S1). Both proteins bound IgG1 Fc with increased affinity compared to the same protein expressed in the WT cell line; however, the F158 allotype bound with a 13-fold weaker affinity than V158 (Fig. S3). While both allotypes gain increased affinity with oligomannose glycans, the gain in affinity by V158 is more substantial than F158 (3.1× *versus* 1.6×; Table S2). This result indicates that the factor causing weaker F158 binding is also present in the oligomannose N-glycoform.

The F158-encoding polymorphism introduces an aromatic residue at a position not found in V158. Aromatic residues form strong interactions through CH-π dispersive forces with carbohydrate residues (31). Thus, it is possible that F158 introduces an inhibitory intramolecular contact. We enzymatically truncated the N-glycans to a single GlcNAc residue and again found that F158 bound with six-fold weaker affinity than V158. Lastly, we prevented N162 glycosylation through the S164A mutation to disrupt the NVS glycosylation sequon. Again, the F158 allotype bound IgG1 Fc with six-fold weaker affinity than V158. When comparing to the native form of the receptor, both allotypes possess nearly identical relative affinity of the single GlcNAc and S164A forms (Table S2). These data demonstrate that the weaker F158 binding affinity is independent of N-glycan composition and the presence of the N162 glycan.

### CD16a F158 exhibits slower binding kinetics

**Figure 4:**
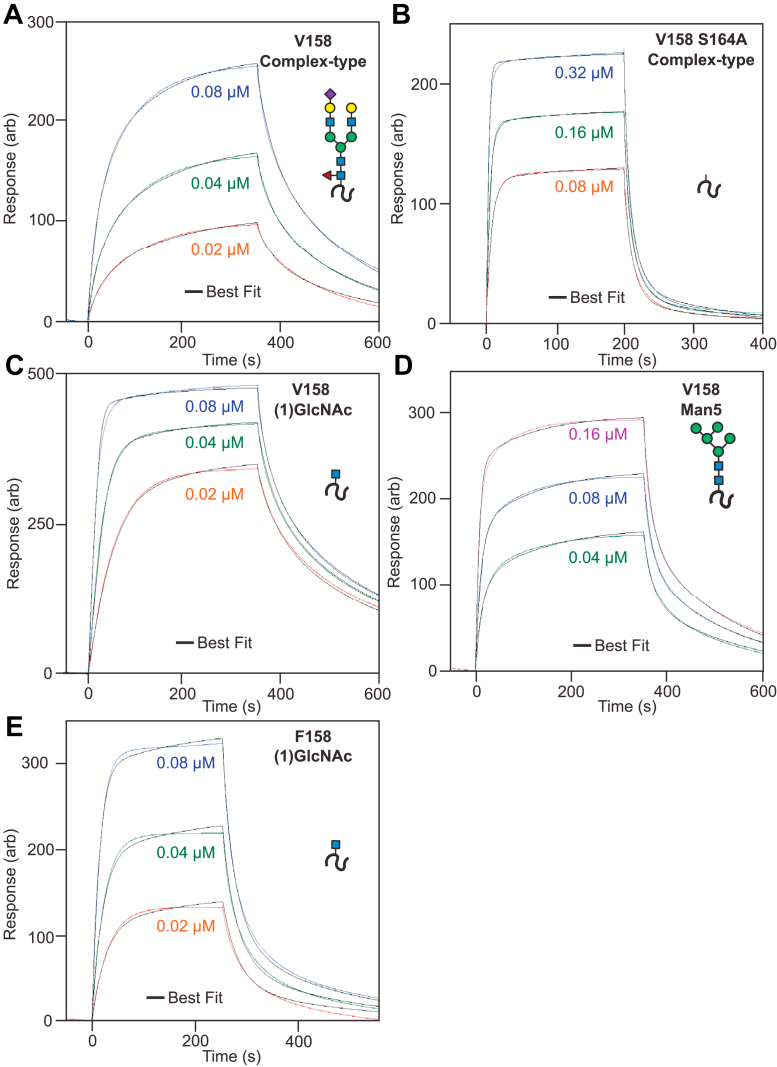
**Binding kinetics show different rates for the glycoform and allotype variants.** IgG1 Fc binding (*A*) CD16a V158 with complex-type N-glycans, (*B*) CD16a V158 S164A with complex-type N-glycans, (*C*) CD16a V158 with truncated (1)GlcNAc N-glycans, or (*D*) with Man5 glycans. *E*, CD16a F158 with (1)GlcNAc N-glycans. Sensorgrams were fitted with a two-state kinetic model (*black line*). *Cartoons* show the expected N-glycan at the N162 site. IgG1, immunoglobulin G1.

A detailed analysis of the binding curves revealed differences in binding rates. We fitted a two-state binding model to the sensorgrams that revealed major (*k*_a_1, *k*_d_1) and minor (*k*_a_2, *k*_d_2) events, with the major event dominating the observed data (Fig. 4, Table 2). The fitting errors were less than 2% in all instances. Dissociation constants resulting from the kinetic fits were lower than those found with fitting the equilibrium responses; however, the relative differences between forms were preserved. Fitting a 1:1 binding model revealed rates highly comparable to the major event in the two-state model, however with slightly increased residuals due to an inability of this model to accommodate slight nonlinearity at the later timepoints of the association curves. This nonlinearity may be due to reversible nonspecific interactions with the chip surface or a slow conformational rearrangement once bound.

**Table 1:** CD16a-binding affinities from at least five individual measurements (Ave ± StDev)

Allotype	N-Glycoform	*K*_D_ (nM)	Error (nM)
V158	Complex	270	40
V158	Man5	86	13
V158	(1)GlcNAc	22	3
V158	S164A	230	40
F158	Complex	1660	150
F158	Man5	1070	120
F158	(1)GlcNAc	126	16
F158	S164A	1470	100

**Table 2:** Kinetic parameters from a two-state binding model

Protein	N-glycoform	*k*_a_1 (1/Ms)	*k*_a_2 (1/s)	*k*_d_1 (1/s)	*k*_d_2 (1/s)	*K*_D_ (nM)	Error			
							*k*_a_1 (1/Ms)	*k*_a_2 (1/s)	*k*_d_1 (1/s)	*k*_d_2 (1/s)
V158	Complex-type	1.7E+05	6.1E-03	2.5E-02	6.6E-03	76	0.65%	0.67%	0.84%	0.25%
V158	Man5	7.1E+05	4.9E-03	7.1E-02	6.0E-03	55	0.72%	0.33%	0.78%	0.17%
V158 S164A	Complex-type	1.4E+06	1.4E-03	1.8E-01	7.8E-03	109	0.82%	0.64%	0.85%	0.51%
V158	(1)GlcNAc	2.5E+06	3.2E-03	4.0E-02	4.7E-03	9.2	0.81%	0.90%	1.01%	0.52%
F158	(1)GlcNAc	1.1E+06	1.8E-03	7.9E-02	5.0E-03	52	1.81%	0.91%	1.94%	0.72%

We observed differences between CD16a V158 binding and binding of the V158 S164A variant, both with complex-type N-glycans but the latter lacking the N162 glycan. The V158 S164A variant bound with an eight-fold increased on rate and a seven-fold increased off rate. These data indicate that the N162 glycan slows complex formation, though the resulting complex exhibits a slower dissociation rate. Shortening the V158 N-glycan with the Man5 glycoforms increased the binding rate by 4.2-fold and also increased the dissociation rate by 2.8-fold. The F158 association proved too weak and too fast for reproducible measurements of the binding kinetics.

The CD16a V158 variant with the truncated (1)GlcNAc N-glycans revealed a 15-fold faster association rate than the complex-type glycoform; however, the dissociation rates were similar (1.6-fold difference). These results support the evidence from the S164A example above that the extended N-glycan slowed the association rate. In contrast to S164A, however, the truncated (1)GlcNAc proved sufficient to stabilize the complex.

The increased binding affinity for the CD16a F158 allotype with truncated (1)GlcNAc N-glycans provided the opportunity to measure binding kinetics and compare V158 and F158 directly. The CD16a F158 (1)GlcNAc protein exhibited an on rate that was 2.3-fold slower than the CD16a V158 (1)GlcNAc glycoform and a dissociation rate two-fold faster. Thus, the presence of F158 slowed the binding and increased the rate of complex dissociation. These differences are consistent with a greater conformational heterogeneity of the F158 allotype.

### NMR shows CD16a structural differences

We evaluated CD16a V158 and F158 using solution NMR spectroscopy to identify possible structural differences. Glycosylated proteins represent a substantial challenge for solution NMR spectroscopy, largely because the common expression host for NMR, *Escherichia coli*, does not N-glycosylate. We expressed CD16a in the same human cell line used for the binding affinity measurements (HEK293F). These cells glycosylate appropriately but do not allow uniform amino acid labeling from metabolic precursors. Instead, we first supplemented the expression medium with [^15^N]-phenylalanine, an essential amino acid that does not scramble under the conditions used for expression.

**Figure 5:**
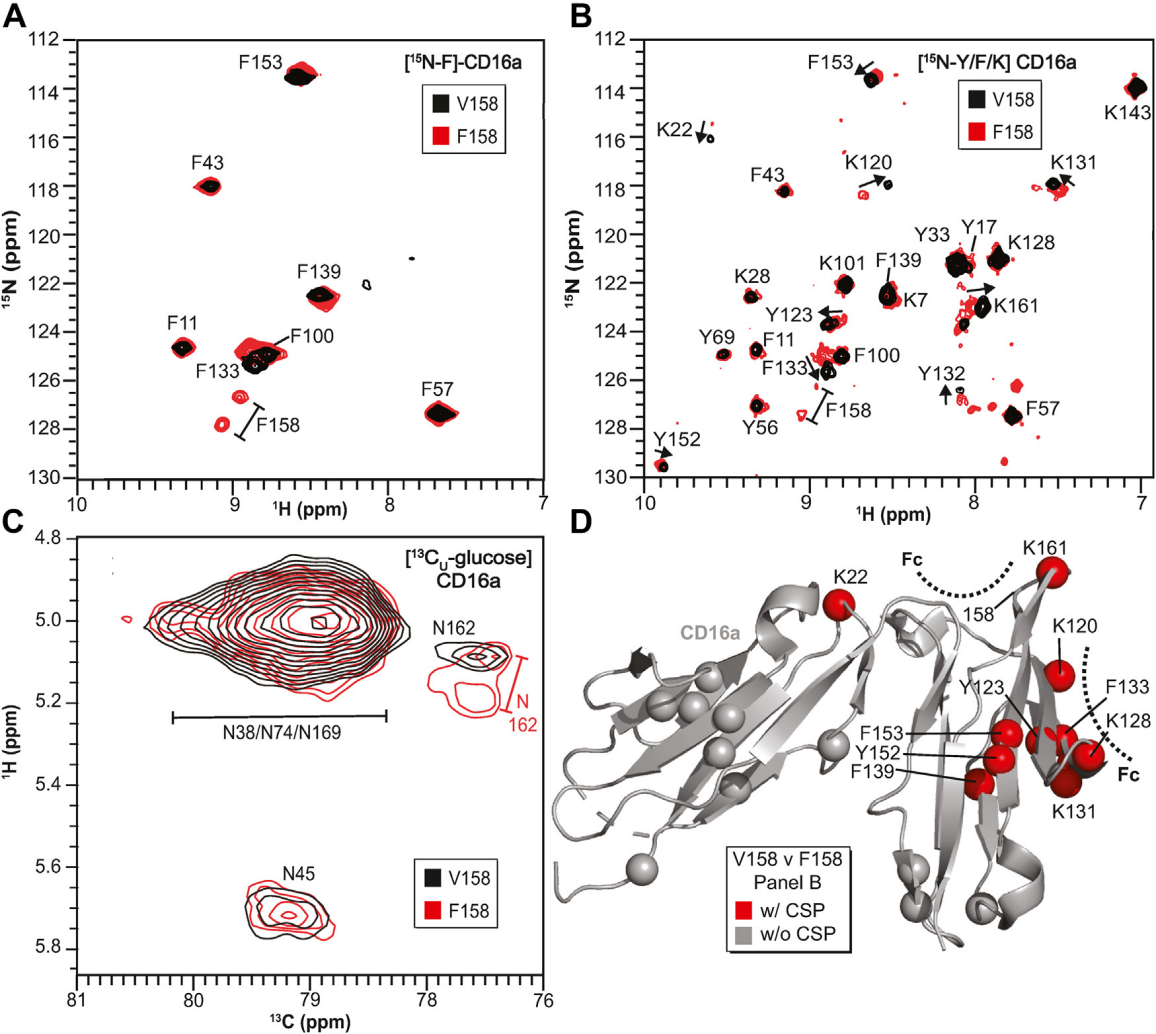
**NMR spectroscopy of the CD16a allotypes.***A*, overlay of HSQC-TROSY spectra for [^15^N-phenylalanine]-labeled CD16a collected at 30 °C and 21.1 T. *B*, overlay of HSQC-TROSY spectra for [^15^N-phenylalanine/lysine/tyrosine]-labeled CD16a. *C*, overlay of ^1^H-(^13^C direct observe) spectra of [^13^C-glucose]-labeled CD16a showing the region corresponding to the (1)GlcNAc ^1^H_1_-^13^C_1_ correlation. *D*, mapping residues with different positions (*red spheres*) and similar positions (*gray spheres*) from panel (*B*) onto a structure of CD16a. The IgG1 Fc–binding interface is shown with *dashed lines*. IgG1, immunoglobulin G1.

An HSQC-TROSY spectrum of [^15^N-phenylalanine]-CD16a V158 showed seven clear peaks corresponding to seven phenylalanine residues (Fig. 5*A*). A similar spectrum of [^15^N-F]-CD16a F158 showed nine peaks, seven of these similar to V158 peaks. The two remaining peaks are likely due to F158. It is curious to note that the F158 N-H correlation revealed two peaks, indicating the presence of two different conformations exchanging slowly on the NMR timescale. It is not known if the V158 N-H correlation likewise samples two conformations; the nitrogen atom from [^15^N]-valine is metabolically scrambled to other amino acids during expression and a V158 peak was not identified (data not shown). It is also notable that the F133 and F153 peaks do not perfectly overlay in spectra of the [^15^N-phenylalanine]-CD16a allotypes, instead showing chemical shift perturbations.

We next probed chemical shift perturbations in other amino acid residues by adding [^15^N]-lysine, [^15^N]-phenylalanine, and [^15^N]-tyrosine to the expression medium. Lysine and tyrosine likewise did not scramble. We were able to match each peak to a residue using the assignment of a related receptor by Kato *et al.* (32). In total, these spectra showed ten residues with chemical shift perturbations, notably at F133, K131, F153, and K161 (Fig. 5*B*).

The N162 glycan is also in the general vicinity of these perturbed residues. N-glycans can be labeled by supplementing the expression medium with [^13^C]-glucose, which is then incorporated into each N-glycan sugar and alanine methyls (33, 34). One unique N-glycan proton-carbon correlation provides unique insight into each individual N-glycan at the point of attachment: the ^1^H_1_-^13^C_1_ correlation on the (1)GlcNAc residue that is covalently bonded to the N-glycosylated asparagine residue (35). NMR observation of this correlation provides a fingerprint of the glycoprotein N-glycans with one peak expected for each N-glycan. We previously assigned the peaks for the CD16a V158 allotype, including a distinct single peak for the N162 glycan (36).

Comparing the CD16a V158 and F158 N-glycan peaks in NMR spectra showed a high degree of similarity for the N38, N45, N74, and N169 peaks (Fig. 5*C*). Surprisingly, CD16a F158 showed two peaks for the N162 glycan, indicating the presence of conformational exchange. One of these peaks overlayed with the single CD16a V158 N162 peak, indicating that CD16a F158 populates conformations either not visible or minimally populated with V158.

Individual atoms that demonstrated a chemical shift perturbation between V158 and F158 clustered on a structural model of the CD16a V158 extracellular domain (Fig. 5*D*). This hotspot is directly adjacent to the antibody-binding interface. Surprisingly, we also identified shifts in these same residues upon removing the N162 glycan through a S164A mutation to disrupt the N162-glycosylation sequon (V158 S164A; Fig. S4). Furthermore, these peaks appeared in an intermediate position between the same peak in spectra of the V158 and F158 allotypes. One exception is K161, which showed a stronger peak in the V158 S164A spectrum. These peak behaviors are consistent with differences in backbone conformations for the loop containing the N162 glycosylation site, and factors affecting conformations include sidechain packing at the 158 position as well as presence of the N-glycan.

It is notable that the NMR experiments revealed differences with unliganded CD16a. We were unable to identify CD16a residues in a complex with Fc, presumably due to the relatively short lifetime of the bound state (data not shown). Data from the unliganded receptors reflects the unbound state and the slower association of the F158 allotype is consistent with greater conformational heterogeneity observed by NMR and thus a model with increased conformational sampling in the unbound F158 reducing the binding rate and thus affinity.

## Discussion

These experiments identified structural, kinetic, and functional differences in the two predominant CD16a allotypes that differ by a single amino acid residue. Multiple aspects of this study are notable. The F158 and V158 allotypes bind ligand with different affinities and at different rates, despite the high degree of sequence similarity. F158 appeared to bind slower and form a less stable complex compared to V158. The lower stability, and thus lower affinity, may be explained by structural modeling that the indicated F158 residue displaces local amino acid residues due to a greater size of the F sidechain (Fig. 1). These slight structural differences are potentially sufficient to destabilize regions outside of the residues that directly contact F158. Indeed, NMR revealed structure differences in the F158 and V158 allotypes that extended far from residue 158. NMR spectra revealed the presence of at least two conformations sampled by the F158 N162 glycan at the point of attachment. Only one conformation was observed for V158. Furthermore, the F158 backbone amide likewise sampled two conformations. The structural changes were not isolated to F158 and residues directly contacting F158, but rather spread throughout the antibody-binding interface.

Previous observations of CD16a isolated from primary human NK cells and monocytes identified the presence of oligomannose N162-glycans (19, 20). These underprocessed forms increased affinity for the V158 allotype (22), though it remained unknown how N-glycan composition affected F158. We determined that the composition of the N162 glycan did not affect F158 and that overall glycan composition had similar impact in both allotypes. The relative affinity differences due to N-glycan composition proved highly similar for V158 and F158 (Table S2). It should be noted that V158 saw a greater affinity increase with Man5 N-glycans (Table 1). Small N-glycans increased the affinity for both allotypes ((1)GlcNAc > Man5 > complex type), thus the mechanism underlying the affinity increases appears to be shared by both proteins. These results are important to interpret the effect of N-glycan composition, determined by profiling FcγR-expressing cells, on antibody binding affinity, and is particularly true for the more common F158 allotype.

Removing or shortening the N162 glycan increased binding rates. This observation is consistent with previous computational modeling of the CD16a–IgG1 complex predicting that N162 glycan conformational heterogeneity negatively affects binding affinity (26, 37). Our data indicate the distal portions of the N-glycan slow binding, with shorter N-glycans binding with increased rates as was evident for the (1)GlcNAc glycoform that bound more quickly than the protein with complex-type glycans. This observation, together with the NMR data, support a model in which one CD16a conformation binds Fc. The presence of an N162 glycan likely exacerbates the impact of multiple slowly interconverting conformations, only one of which can bind Fc. The lack of glycosylation of the N162 site in the V158 S164A mutant showed the fastest association rate and also a single intense K161 peak in NMR. It is possible that the increased binding rate for the V158 S164A mutant is due to sampling fewer conformations or more rapidly sampling conformations, thus overcoming a kinetic barrier to binding. While the distal portions of the N-glycan largely appear dispensable for high affinity binding, they do promote receptor/antibody stability to a greater extent than (1)GlcNAc (1.6-fold), Man5 (2.9-fold), and S164A (7.1-fold). These data indicate an N162 glycoform for optimal binding may be found at a size between (1)GlcNAc and Man5.

The prevalence of the F158 allotype in the human population suggests increased fitness is associated with at least one copy of the weaker binding allele. Unfortunately, patients expressing F158 show decreased responses to many antibody-based therapeutics, though advances in antibody engineering may surmount this barrier (38). Here, we report a study of the differences in the common CD16a allotype. While the effects of this difference are known in a therapeutic context, the underlying mechanism was unknown. Here, we provide detailed analysis of the differences caused by the 158 allotype and hint at a possible mechanism of action. This work provides the foundation of knowledge necessary to begin developing improved therapeutic antibodies. Lastly, increasing the antibody-binding affinity of the F158 allotype may be achieved by developing reagents to specifically truncate the F158 N162 glycan or antibody Fc designs that accommodate the unique structural features including multiple conformations of the antibody-binding interface.

## Experimental procedures

### Materials

All materials were purchased from Millipore-Sigma unless otherwise noted.

### Protein expression for binding affinity measurements

The V and F CD16a allotypes were cloned into the pGen2 plasmid downstream of and in frame with DNA encoding the 8x-histidine tag, GFP, and tobacco etch virus (TEV) protease digestion site as described (39). Human IgG1 Fc (residues 216–447) was expressed from a plasmid previously described (40). All proteins were expressed through transient transfection of either HEK293F (Life Technologies) or HEK293S (Gnt1-) cells (30). Unless otherwise specified, cells were grown in FreeStyle293 medium (Life Technologies) supplemented with ExCell (Sigma Aldrich) media, 10% of total volume, on a shaker (ATR Biotech) at 125 RPM with 8% CO2 and 80% humidity at 37 °C. Cell densities were 3.0 × 10^6^ live cells/ml at the time of transfection. We added 2.5 μg/ml DNA and 10 μg/ml PEI (40 KDa). Following a 24 h incubation, cultures were diluted 1:1 with the same media containing 4.4 mM valproic acid. Culture supernatant was harvested after 5 days by centrifugation at 1000*g* for 5 min. CD16a was purified using a Ni-NTA column (Qiagen), while Fc was purified using a Protein A-Sepharose column. All proteins were stored in a buffer containing 25 mM 3-(N-morpholino) propanesulfonic acid (MOPS), 0.1 M sodium chloride, pH 7.2.

### Endoglycosidase F1 digestion

EndoF1 was expressed with *E. coli* and coupling at a density of (15 mg protein per ml of resin) to AminoLink Coupling Resin (Thermo Scientific) following manufacturers’ protocol as previously described (26) in a buffer containing 0.1 M sodium phosphate, 0.15 M NaCl, pH 7.2. CD16a was digested at a ratio of 1 mg per 50 μl of prepared resin overnight at room temperature. Digestion was analyzed using SDS-PAGE.

### Surface plasmon resonance

All affinity measurements were performed with a Biacore T200 (GE Life Sciences) using amine coupling to a CM5 chip. Fc was coupled at 1 μg/ml for saturation experiments. Several different CD16a concentrations, ranging between 0.01 μM and 20.48 μM, was flowed over the chip surface at a rate of 10 μl/min. CD16a was diluted in running buffer containing 20 mM MOPS, 100 mM sodium chloride, 1 μM bovine serum albumin, and 0.05% P20 surfactant (GE Life Sciences), pH 7.4. Contact and dissociation times of at least 300 s were used for all proteins. The chip was regenerated after each step with 100 mM glycine, pH 3.0 for 30 s.

### Kinetic measurements

All kinetic measurements were performed in the same manner as surface plasmon resonance unless specified. The chip was coupled in lanes 2 and 4 at 0.33 μg/ml. Protein concentrations ranged from 0.04 μM and 5.12 μM were flowed over the chip at 60 μl/min with a contact time of 30 s and a dissociation time of 250 s. Bovine serum albumin was not included in the running buffer.

### Protein expression for NMR

Freestyle293 medium without amino acids or glucose or osmolarity adjustment was purchased (Life Technologies). This base medium was supplemented with all amino acids at 100 mg/l, with the exception of glutamine, phenylalanine, lysine, and tyrosine. Glutamine (1 g/l) and glucose (3 g/l) were added. Next, 100 mg/l [^15^N]-lysine, [^15^N]-phenylalanine, and [^15^N]-tyrosine were added. For NMR experiments utilizing ^13^C, the medium was supplemented with 3 g/l [^13^C_U_]-glucose. pH was adjusted to 7.20 before adjusting osmolarity with 5 M NaCl to 260 to 280 mOsm/kg, followed by a final adjustment of pH to 7.20. The labeled media was passed through a sterile 0.2 μm aPES membrane (Fischer Scientific) and stored at 4 °C.

Isotope-labeled CD16a for NMR was expressed and diluted with the labeling culture medium using the transfection, dilution, and purification methods stated above. TEV was expressed from a plasmid provided by Dr Kelley Moremen (UGA) in *E. coli* and purified with a Ni-NTA resin. GFP-CD16a was buffer exchanged with 50 mM Tris–HCl, 0.5 mM EDTA, 1 mM DTT, pH 8.0, then digested with 1:25 (w:w) GFP-CD16a:TEV ratio overnight at room temperature with end-over-end mixing. The following day, the mixture was passed over a Superdex 75 column (GE Health Sciences). Fractions containing CD16a were identified with SDS-PAGE and were subsequently pooled. The protein was buffer exchanged into a buffer containing 20 mM sodium phosphate, 100 mM potassium chloride, 5% D_2_O, 0.05 mM trimethylsilylpropanesulfonate and concentrated to approximately 150 μl using Amicon Ultra 0.5 ml 10 kDa cellulose cartridge.

### NMR spectroscopy

NMR spectra were collected at a 30 °C sample temperature on a 21.1 T spectrometer equipped with a Bruker NEO console and 5 mm TXO cryoprobe. Direct ^13^C-observed experiments were collected with the hxinepph pulse sequence, with 16,384 and 128 complex points in the direct and indirect dimensions, respectively. The Bruker hsqcetfpf3gpsi2 pulse sequence was used to collect ^1^H-^15^N correlations with 1024 and 256 complex points in the direct and indirect dimensions, respectively. Spectra were processed with a sine-squared and 40 Hz exponential multiplier line broadening function applied in the direct dimension and a sine function with a 10 Hz exponential multiplier in the indirect dimension. Spectra were processed in NMRPipe (41) and analyzed in NMRViewJ (42).

### Mass spectrometry

Glycopeptides for the F158 and V158 proteins were prepared as previously described (19). Glycopeptides from each sample were enriched using TopTip PolyHydroxyethyl A (HILIC) 10 μl tips (Glygen) following manufacturer guidelines. Samples were eluted from the resin three times into the same receptacle with 10 μl elutions of 15 mM ammonium acetate, 10% acetonitrile (v/v) pH 3.5. The resulting elutions were lyophilized and then desalted with C18 ZipTips (Millipore) according to the manufacturer’s protocol. The samples were resuspended in 20 μl of deionized water. Each sample (0.5 μl) was loaded into an EASY nLC-1200 LC system with PepMap nanocolumn (ThermoFisher) and a Nanospray FlexIon source (ThermoFisher) and analyzed with a Q Exactive Plus Hybrid Quadrupole-Orbitrap Mass Spectrometer (ThermoFisher). Liquid chromatography and mass spectrometry were performed as described (43). The generated RAW files were initially analyzed using Byonic (ProteinMetrics). N162 N-glycans were manually evaluated by comparing retention time and analyzing the MS2 spectra with XCaliber Qual Browser (ThermoFisher; see Fig. S5).

## Data availability

MS data were deposited in the MASSIVE database (http://massive.ucsd.edu) under accession MSV000089756.

## Supporting information

This article contains supporting information (19, 20).

## Conflict of interest

The authors declare no conflicts of interest.
