# Supplementary material for: The weaker-binding Fc γ receptor IIIa F158 allotype retains sensitivity to N-glycan composition and exhibits a destabilized antibody-binding interface
Source: J Biol Chem. 2022 Jul 31;298(9):102329. doi: 10.1016/j.jbc.2022.102329 (PMC9436803; doi:10.1016/j.jbc.2022.102329)
Supplement: Supplemental Figures S1–S5 and Tables S1, S2 [file mmc1.pdf]

Supporting Information for:

The weaker-binding Fc γ receptor IIIa F158 allotype retains sensitivity to N-glycan composition and exhibits a destabilized antibody-binding interface

Paul G. Kremer[1] and Adam W. Barb[1,2,3]

Supplemental Table 1. N162 glycan composition of CD16a expressed in HEK293F cells

Allotype	Glycan	MS2	z	Calc $(M+zH)^{z+}$	Obs $(M+zH)^{z+}$	Intensity (arb)	RT	PPM error	Mass error (Da)	Peptide
F	HexNAc(5)Hex(5)Fuc(1)	X	3	927.0392	927.0381	2500	9	1.230	0.00114	GSKNVSSE
F	HexNAc(5)Hex(4)Fuc(1)	X	3	873.0216	873.0211	5300	9	0.607	0.00053	GSKNVSSE
F	HexNAc(5)Hex(3)Fuc(1)	X	3	819.0040	819.0026	2500	9	1.734	0.00142	GSKNVSSE
V	HexNAc(5)Hex(5)Fuc(1)	X	3	960.0620	960.0625	103000	11	-0.479	0.00046	VGSKNVSSE
V	HexNAc(5)Hex(4)Fuc(1)	X	3	906.0444	906.0444	377000	11	0.033	3E-05	VGSKNVSSE
V	HexNAc(5)Hex(3)Fuc(1)	X	3	852.0268	852.0268	182000	11	0.023	2E-05	VGSKNVSSE
V	HexNAc(4)Hex(3)Fuc(1)	X	3	784.3337	784.3337	240000	10	0.000	0	VGSKNVSSE
V	HexNAc(2)Hex(5)	X	3	870.3758	870.3763	117000	15	-0.574	0.0005	CRGLVGSKNVSSE
V	HexNAc(6)Hex(3)Fuc(1)	X	3	919.7200	919.7196	220000	10	0.381	0.00035	VGSKNVSSE
V	HexNAc(3)Hex(3)Fuc(1)	X	2	1074.4572	1074.4572	150000	10	0.019	2E-05	VGSKNVSSE
V	HexNAc(4)Hex(5)Fuc(1)	X	3	892.3689	892.3685	296000	10	0.471	0.00042	VGSKNVSSE
V	HexNAc(4)Hex(4)Fuc(1)	X	3	838.3513	838.3509	215000	10	0.489	0.00041	VGSKNVSSE
V	HexNAc(5)Hex(6)Fuc(1)	X	3	1014.0797	1014.0799	100000	9	-0.247	0.00025	VGSKNVSSE

Supplemental Table 2. CD16a affinities relative to the complex-type glycoform

	Complex-type	Man5	(1)GlcNAc	S164A
V158	1.0	3.4	13	1.1
F158	1.0	1.6	13	1.1

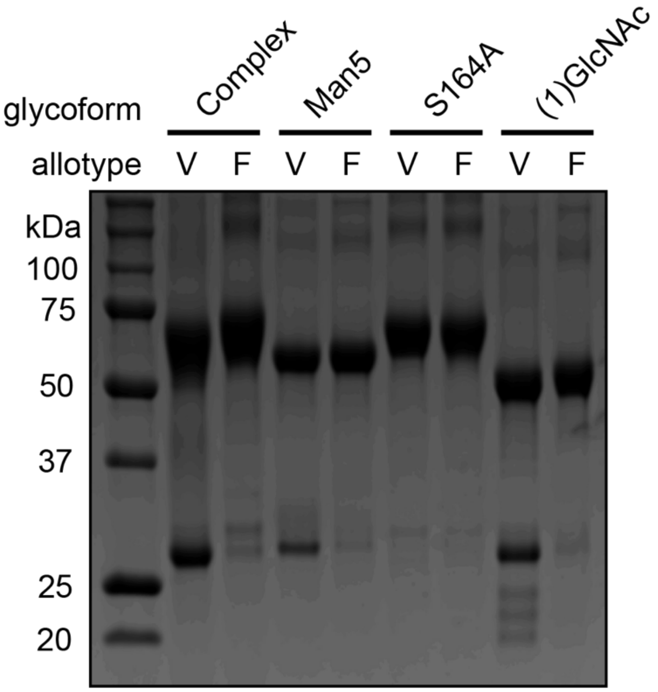

Supplemental Figure 1. SDS-PAGE of GFP-srCD16a fusions. The two different allotypes are expressed with four different glycosylation patterns. Distinct shifts in migration are observed between each type of glycosylated protein.

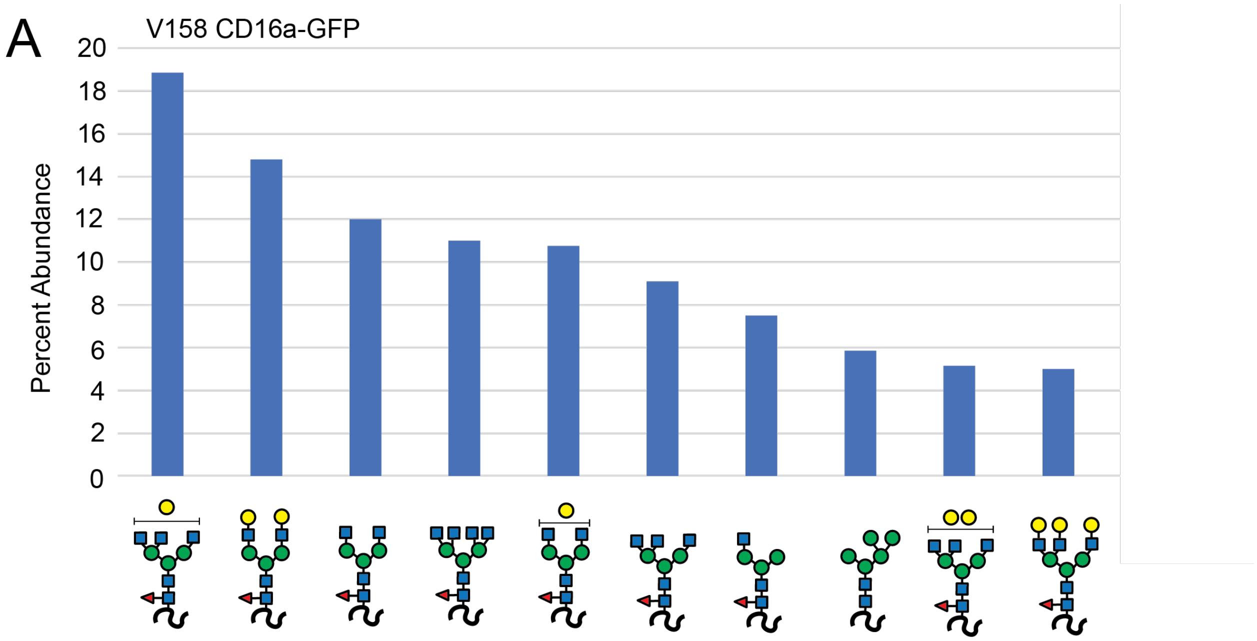

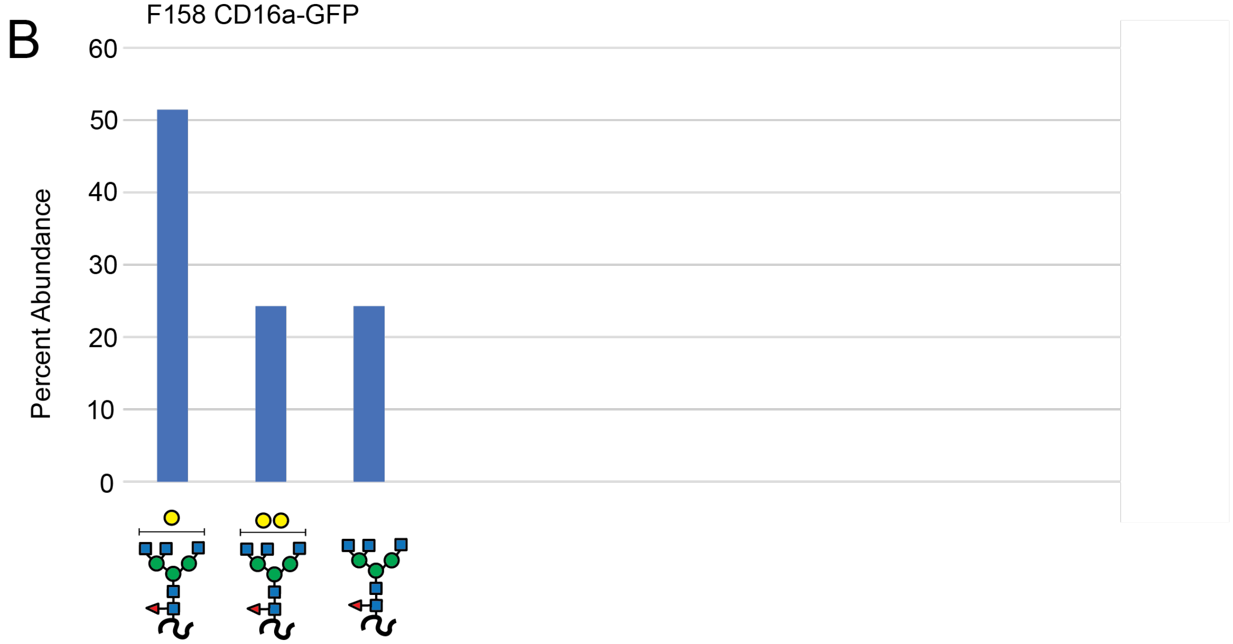

Supplemental Figure 2. Relative abundance of N162 glycans on the V158 and F158 allotypes as determined by glycoproteomics MS. The N162-containing glycopeptide for F158 provides less intense peaks by ESI-MS/MS as our lab previously noted (Mol Cell Proteomics. 2019 Nov;18(11):2178-2190; Mol Cell Proteomics. 2020 Feb;19(2):362-374). Possible N-glycan configurations are shown as cartoons below the chart.

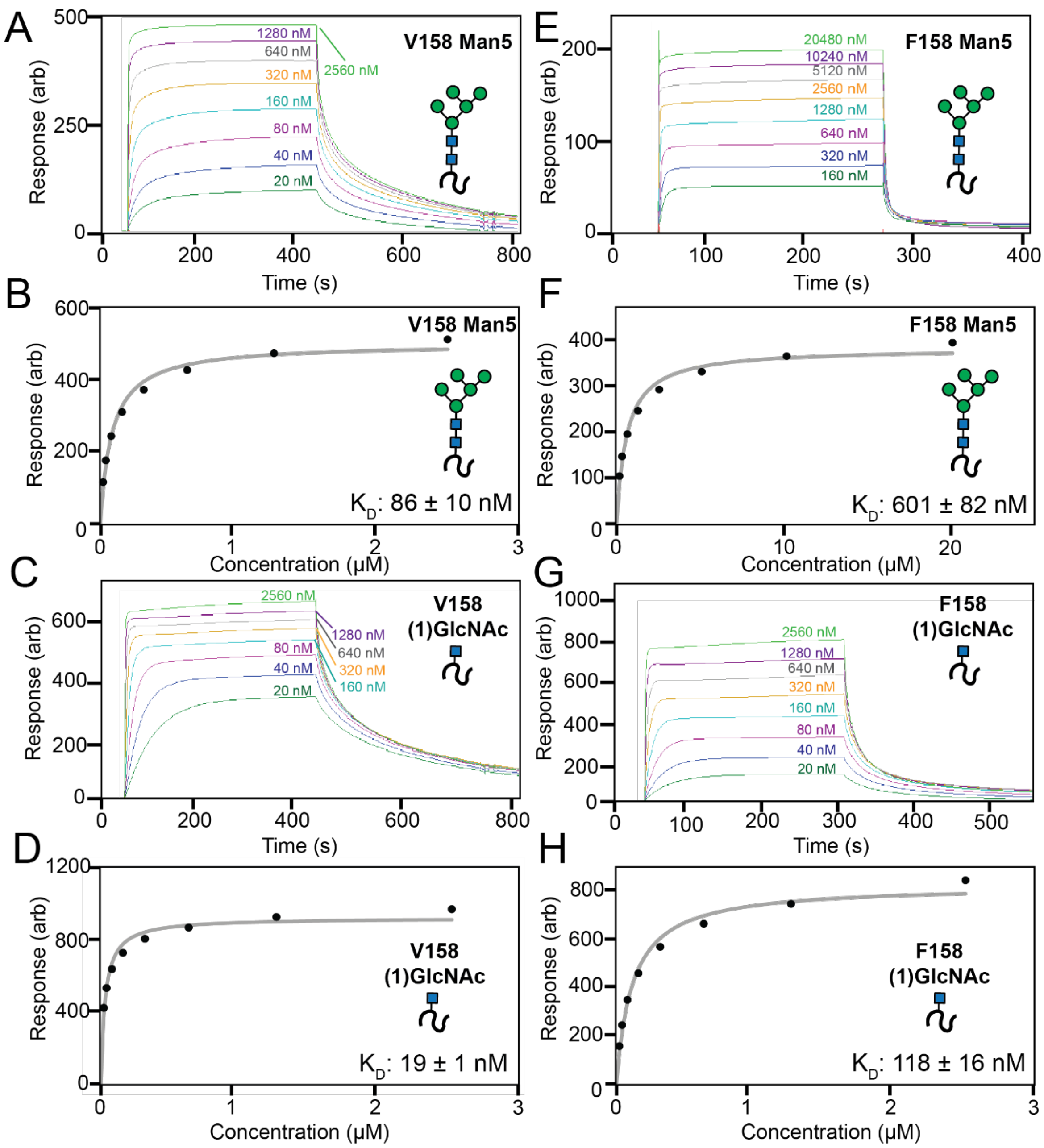

Supplemental Figure 3. Representative surface plasmon resonance (SPR) sensorgrams of CD16a allotypes with either Man5 oligomannose N-glycans (A,E) or (1)GlcNAc N-glycans (C,G). Fits of the dissociation constants and associated errors from equilibrium binding measurements are also shown (B,D,F,H). Cartoons show the expected N-glycan at the N162 site.

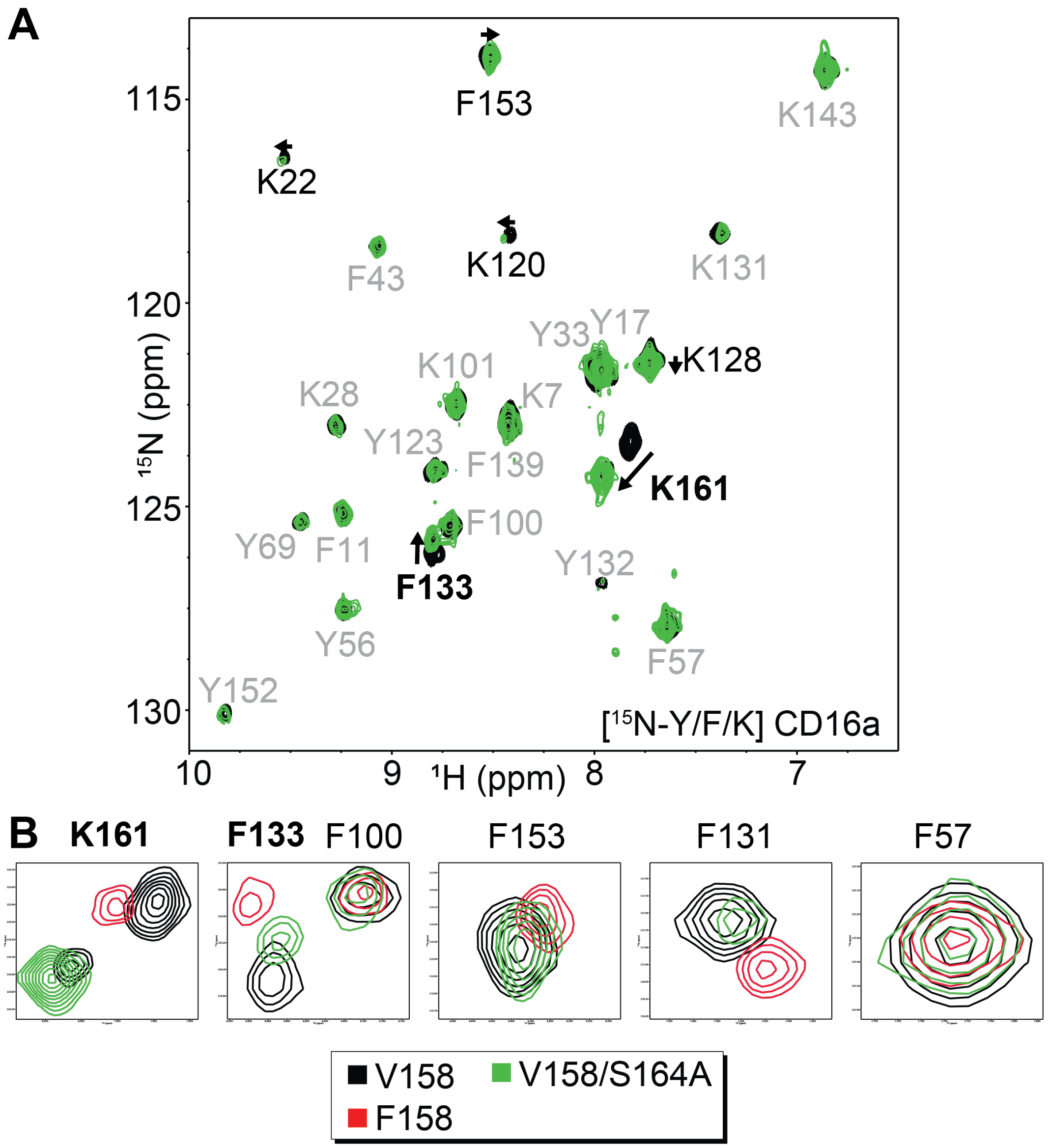

Supplemental Figure 4. (**A**) Overlay of HSQC-TROSY spectra of CD16a V158 with (black) and without (green) the N162 glycan. Residues with large chemical shift perturbations are bolded. (**B**) Highlighted peaks showing differences between the V158, V158/S16A and F158 spectra.

Supplemental Figure 5. Assigned N162 glycopeptide MS2 spectra (following pages)

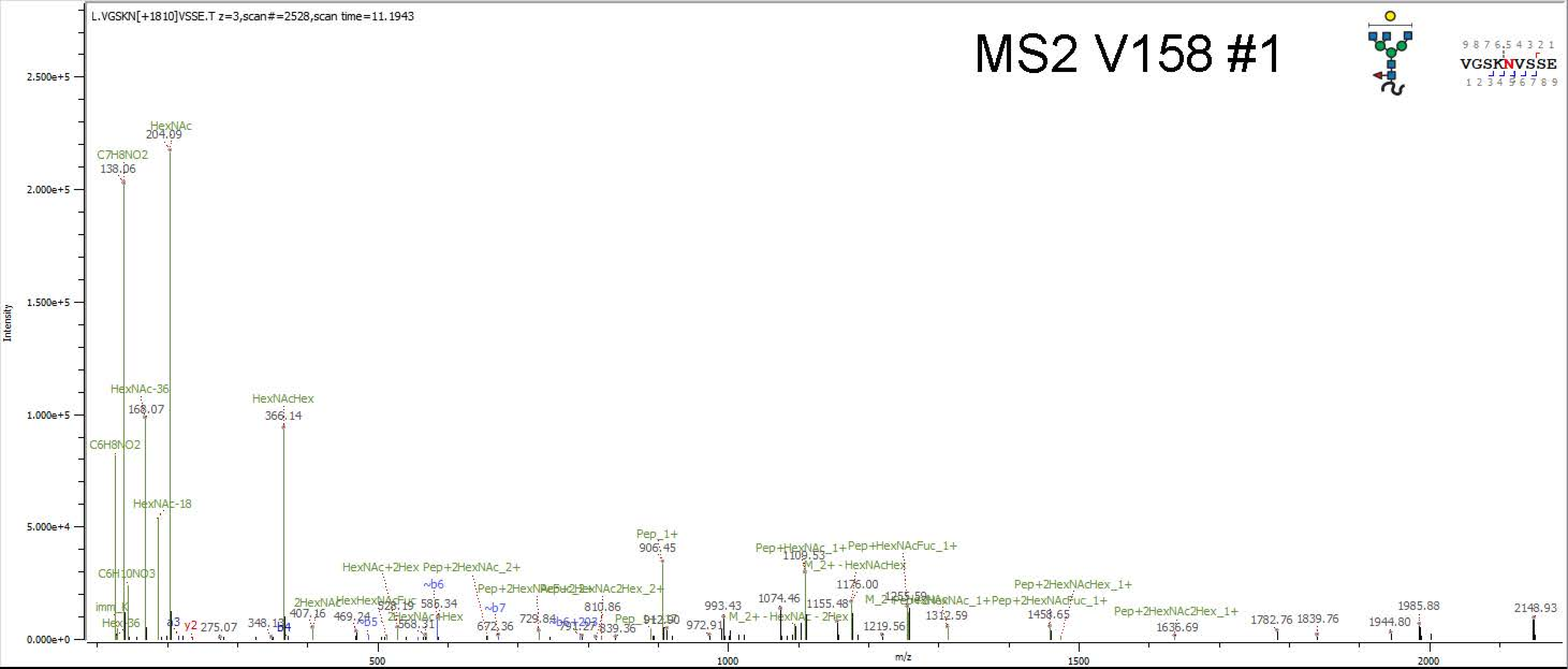

L.VGSKN[+1810]VSSE.T z=3,scan#=2528,scan time=11.1943
MS2 V158 #1
VGSKNVSSE
Intensity
m/z
HexNAc
204.09
C7H8NO2
138.06
HexNAc-36
168.07
HexNAcHex
366.14
C6H8NO2
HexNAc-18
C6H10NO3
imm_K
Hex-36
y2
275.07
348.13
407.16
2HexNAc
HexHexNAcFuc
469.24
528.19
586.34
568.31
~b6
~b5
~b7
HexNAc+2Hex
Pep+2HexNAc_2+
2HexNAc+Hex
672.36
729.84
810.86
839.36
Pep_1+
906.45
972.91
993.43
1074.46
Pep+HexNAc_1+
Pep+HexNAcFuc_1+
1109.53
M_2+ - HexNAcHex
1155.48
1176.00
M_2+ - HexNAc - 2Hex
1219.56
1255.59
1312.59
Pep+2HexNAcHex_1+
Pep+2HexNAcFuc_1+
1458.65
Pep+2HexNAc2Hex_1+
1636.69
1782.76
1839.76
1944.80
1985.88
2148.93
0.000e+0
5.000e+4
1.000e+5
1.500e+5
2.000e+5
2.500e+5
500
1000
1500
2000

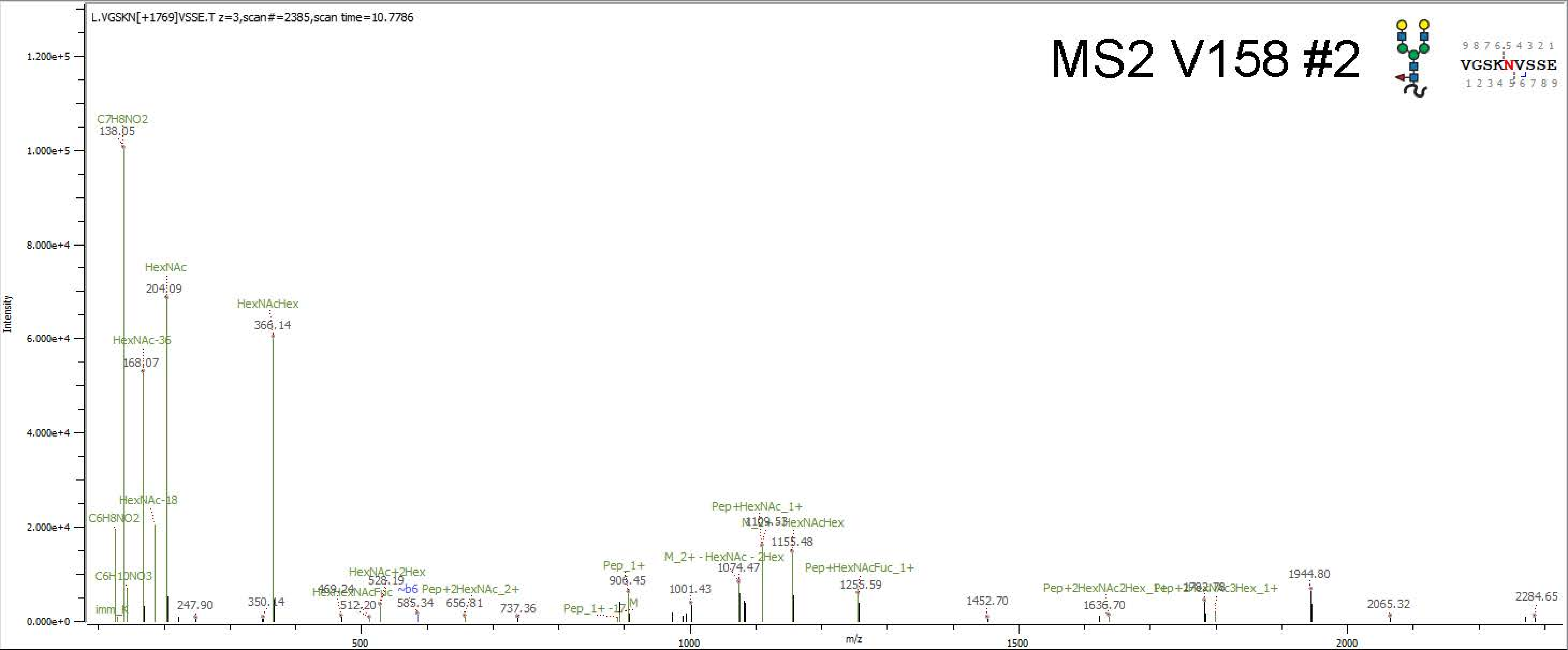

L.VGSKN[+1769]VSSE.T z=3,scan#=2385,scan time=10.7786
MS2 V158 #2
VGSKNVSSE
Intensity
m/z
C7H8NO2
138.05
HexNAc
204.09
HexNAcHex
366.14
HexNAc-36
168.07
HexNAc-18
C6H8NO2
C6H10NO3
imm_K
247.90
350.14
469.24
HexHexNAcFuc
512.20
HexNAc+2Hex
528.19
~b6
585.34
Pep+2HexNAc_2+
656.81
737.36
Pep_1+ -17
M
Pep_1+
906.45
1001.43
M_2+ - HexNAc - 2Hex
1074.47
Pep+HexNAc_1+
1109.53
HexNAcHex
1155.48
Pep+HexNAcFuc_1+
1255.59
1452.70
Pep+2HexNAc2Hex_1+
1636.70
Pep+2HexNAc3Hex_1+
1944.80
2065.32
2284.65

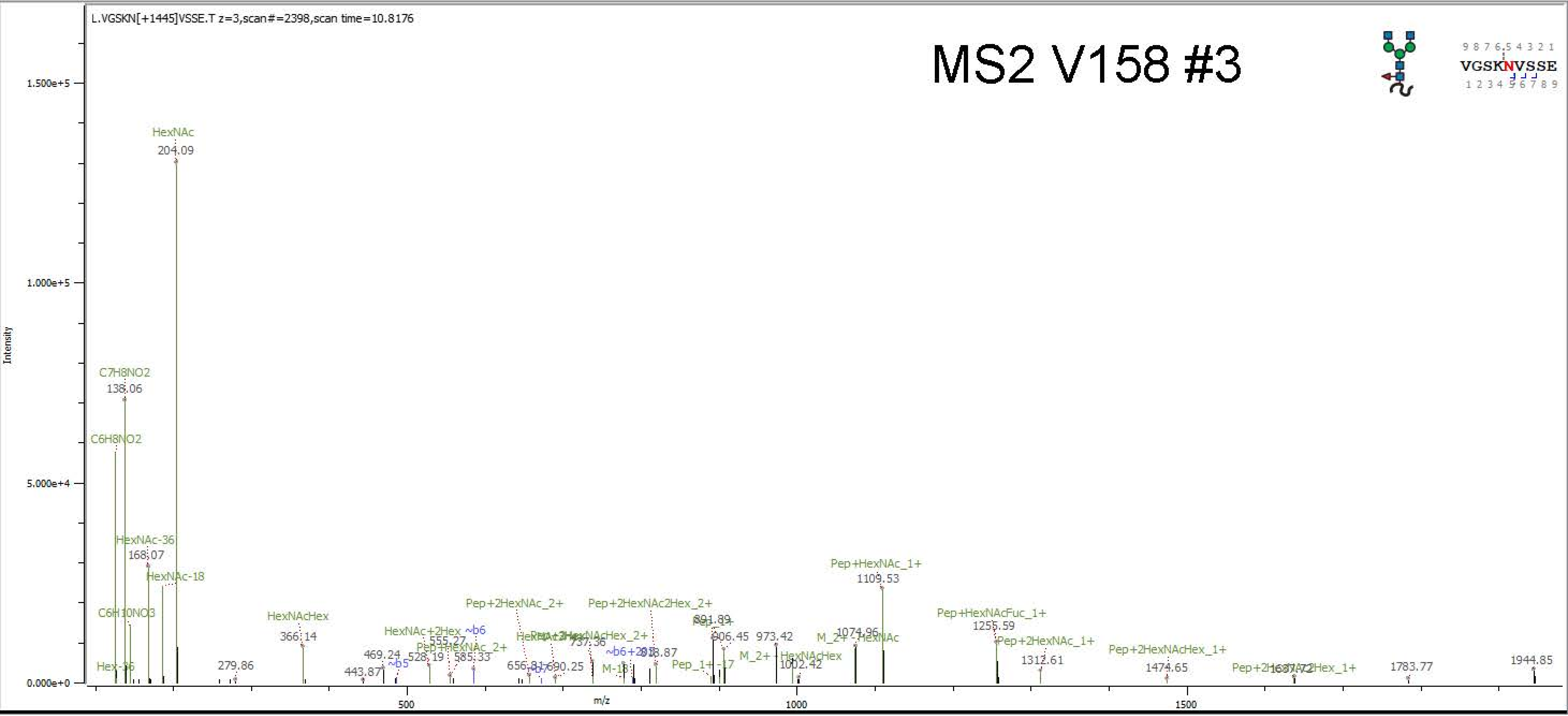
L.VGSKN[+1445]VSSE.T z=3,scan#=2398,scan time=10.8176
MS2 V158 #3
9 8 7 6 5 4 3 2 1
VGSKNVSSE
1 2 3 4 5 6 7 8 9
Intensity
1.500e+5
1.000e+5
5.000e+4
0.000e+0
m/z
500
1000
1500
HexNAc
204.09
C7H8NO2
138.06
C6H8NO2
HexNAc-36
168.07
HexNAc-18
C6H10NO3
Hex-36
279.86
HexNAcHex
366.14
443.87
469.24
~b5
HexNAc+2Hex
~b6
555.27
528.19
585.33
Pep+HexNAc_2+
Pep+2HexNAc_2+
656.81
690.25
737.36
Pep+2HexNAc2Hex_2+
~b6+203
818.87
M-18
891.89
906.45
973.42
Pep_1+
Pep_1+ -17
M_2+
M_2+ -HexNAc
HexNAcHex
1002.42
1074.96
Pep+HexNAc_1+
1109.53
Pep+HexNAcFuc_1+
1255.59
Pep+2HexNAc_1+
1312.61
Pep+2HexNAcHex_1+
1474.65
1783.77
1944.85

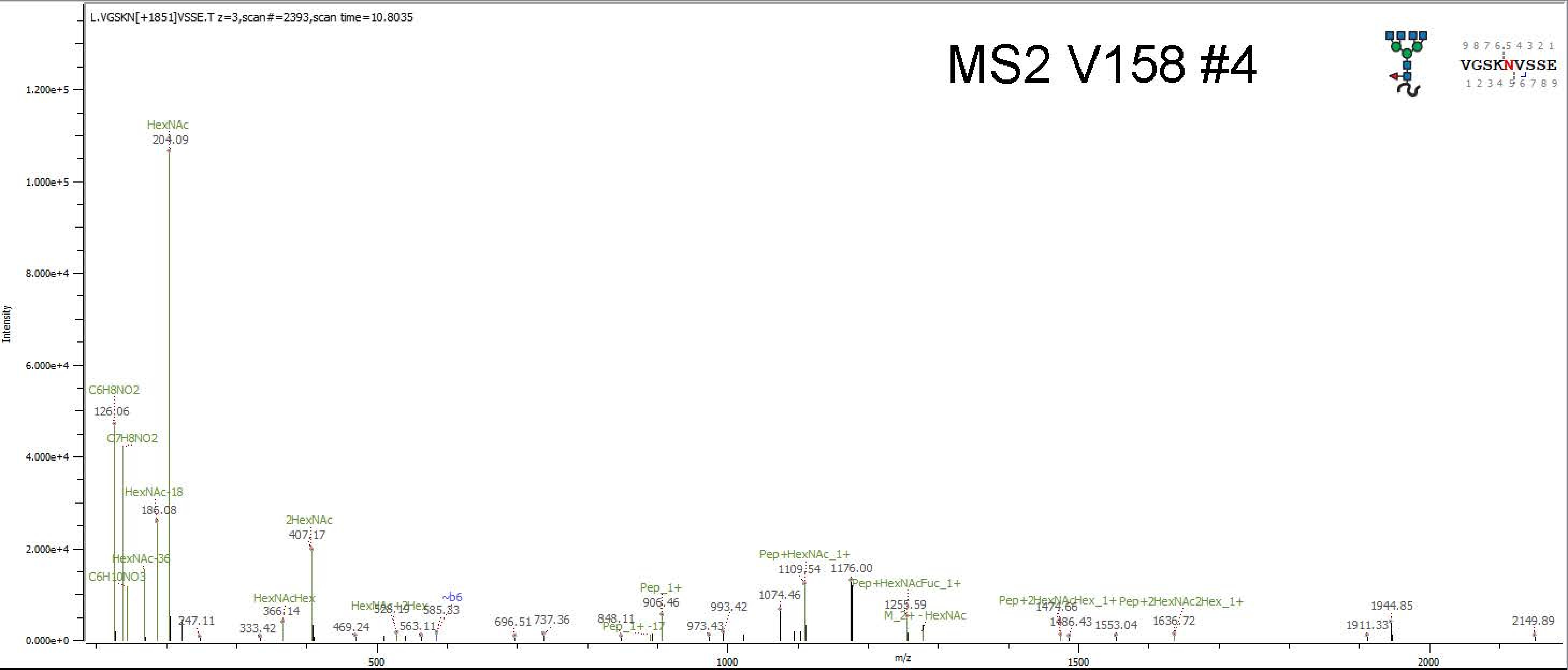

L.VGSKN[+1851]VSSE.T z=3,scan#=2393,scan time=10.8035
MS2 V158 #4
9 8 7 6 5 4 3 2 1
VGSKNVSSE
1 2 3 4 5 6 7 8 9
Intensity
m/z
HexNAc
204.09
C6H8NO2
126.06
C7H8NO2
HexNAc-18
186.08
2HexNAc
407.17
HexNAc-36
C6H10NO3
HexNAcHex
366.14
247.11
333.42
469.24
528.19
563.11
585.33
~b6
696.51
737.36
848.11
Pep_1+ -17
Pep_1+
906.46
973.43
993.42
1074.46
Pep+HexNAc_1+
1109.54
1176.00
Pep+HexNAcFuc_1+
1255.59
M_2+ - HexNAc
Pep+2HexNAcHex_1+
1474.66
1486.43
1553.04
Pep+2HexNAc2Hex_1+
1636.72
1911.33
1944.85
2149.89

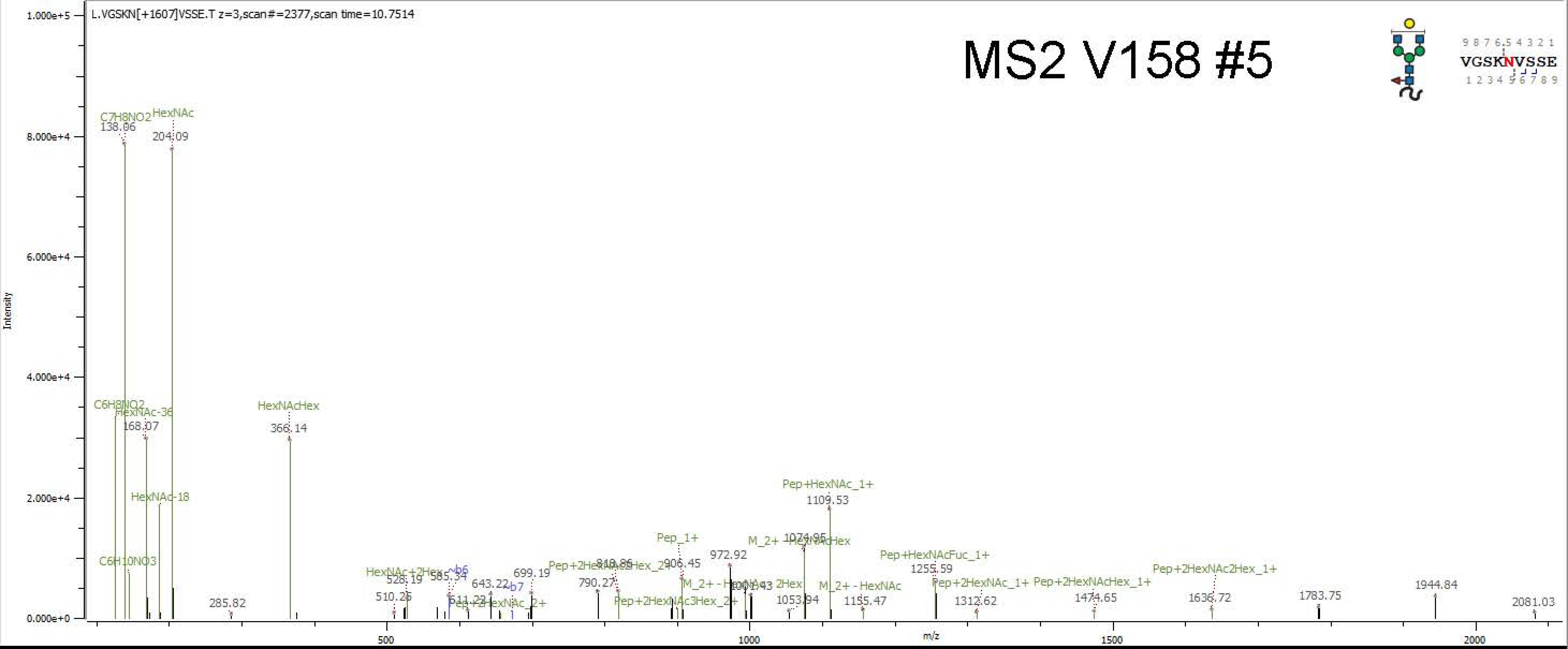

L.VGSKN[+1607]VSSE.T z=3,scan#=2377,scan time=10.7514
MS2 V158 #5
VGSKNVSSE
Intensity
m/z
C7H8NO2
138.06
HexNAc
204.09
C6H8NO2
HexNAc-36
168.07
HexNAcHex
366.14
HexNAc-18
C6H10NO3
285.82
HexNAc+2Hex
510.26
528.19
585.34
611.22
643.22
699.19
Pep+2HexNAc_2+
790.27
Pep_1+
906.45
972.92
M_2+ - HexNAcHex
1074.95
Pep+HexNAc_1+
1109.53
M_2+ - HexNAc
1155.47
1053.94
Pep+HexNAcFuc_1+
1255.59
Pep+2HexNAc_1+
1312.62
Pep+2HexNAcHex_1+
1474.65
Pep+2HexNAc2Hex_1+
1636.72
1783.75
1944.84
2081.03

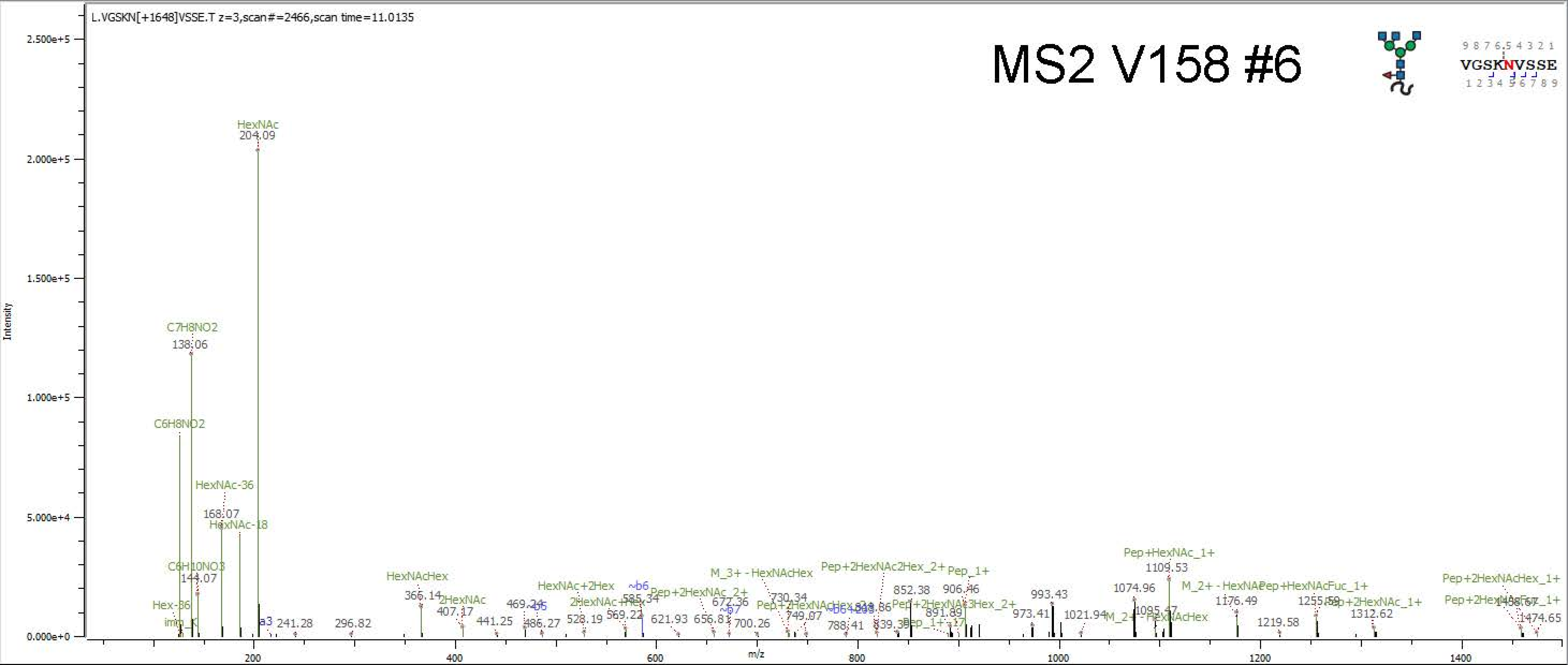
L.VGSKN[+1648]VSSE.T z=3,scan#=2466,scan time=11.0135
MS2 V158 #6
VGSKNVSSE
Intensity
m/z
HexNAc
204.09
C7H8NO2
138.06
C6H8NO2
HexNAc-36
168.07
HexNAc-18
C6H10NO3
144.07
Hex-36
HexNAcHex
366.14
2HexNAc
407.17
HexNAc+2Hex
Pep+2HexNAc_2+
M_3+ - HexNAcHex
Pep+2HexNAc2Hex_2+
Pep_1+
852.38
906.46
993.43
1074.96
Pep+HexNAc_1+
1109.53
M_2+ - HexNAc
Pep+HexNAcFuc_1+
1176.49
Pep+2HexNAc_1+
1312.62
Pep+2HexNAcHex_1+
1474.65
1219.58
1021.94
973.41
891.89
730.34
749.07
788.41
700.26
672.36
656.81
621.93
569.22
585.34
528.19
486.27
469.24
441.25
296.82
241.28

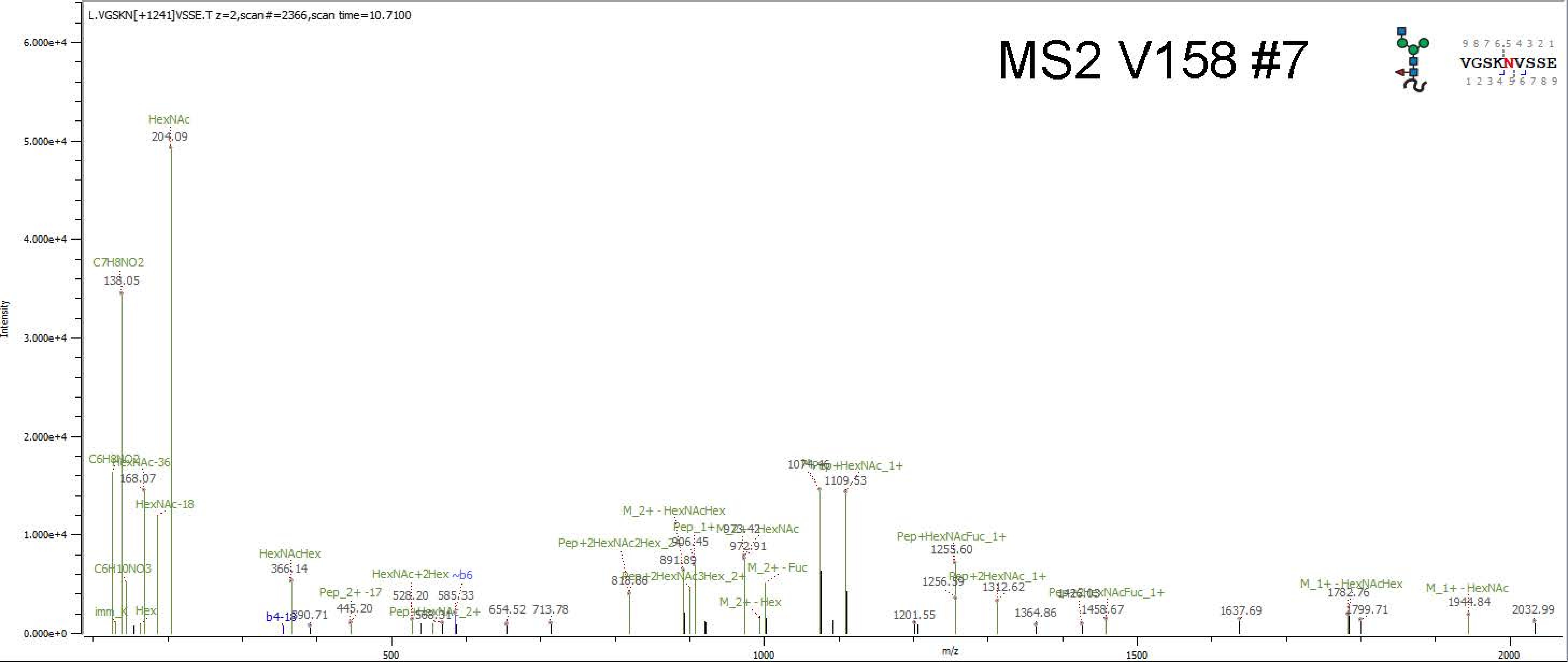
L.VGSKN[+1241]VSSE.T z=2,scan#=2366,scan time=10.7100
MS2 V158 #7
VGSKNVSSE
Intensity
m/z
HexNAc
204.09
C7H8NO2
138.05
HexNAc-36
168.07
HexNAc-18
C6H10NO3
HexNAcHex
366.14
Pep_2+ -17
445.20
HexNAc+2Hex
528.20
585.33
~b6
b4-18
654.52
713.78
Pep+2HexNAc2Hex_2+
818.86
M_2+ - HexNAcHex
906.45
891.89
972.91
M_2+ - Fuc
M_2+ - Hex
Pep+HexNAc_1+
1109.53
Pep+HexNAcFuc_1+
1255.60
1256.59
Pep+2HexNAc_1+
1312.62
1364.86
1201.55
1458.67
1637.69
M_1+ - HexNAcHex
1782.76
1799.71
M_1+ - HexNAc
1944.84
2032.99

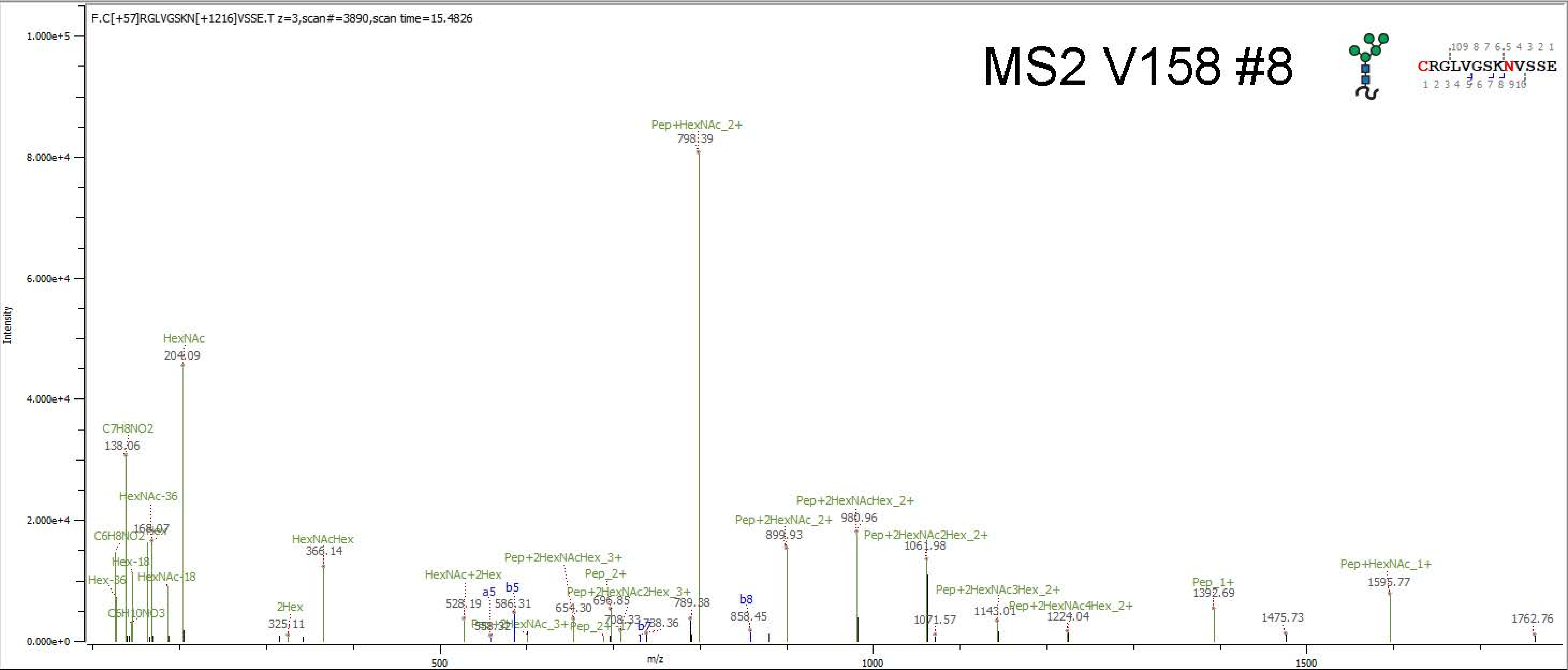
F.C[+57]RGLVGSKN[+1216]VSSE.T z=3,scan#=3890,scan time=15.4826
MS2 V158 #8
CRGLVGSKNVSSE
Intensity
m/z
Pep+HexNAc_2+
798.39
HexNAc
204.09
C7H8NO2
138.06
HexNAc-36
168.07
C6H8NO2
Hex-18
Hex-36
HexNAc-18
C6H10NO3
HexNAcHex
366.14
2Hex
325.11
HexNAc+2Hex
528.19
a5
b5
586.31
Pep+2HexNAcHex_3+
Pep_2+
654.30
696.85
Pep+2HexNAc2Hex_3+
789.38
b8
858.45
Pep+2HexNAc_2+
899.93
Pep+2HexNAcHex_2+
980.96
Pep+2HexNAc2Hex_2+
1061.98
1071.57
Pep+2HexNAc3Hex_2+
1143.01
Pep+2HexNAc4Hex_2+
1224.04
Pep_1+
1392.69
1475.73
Pep+HexNAc_1+
1595.77
1762.76
500
1000
1500

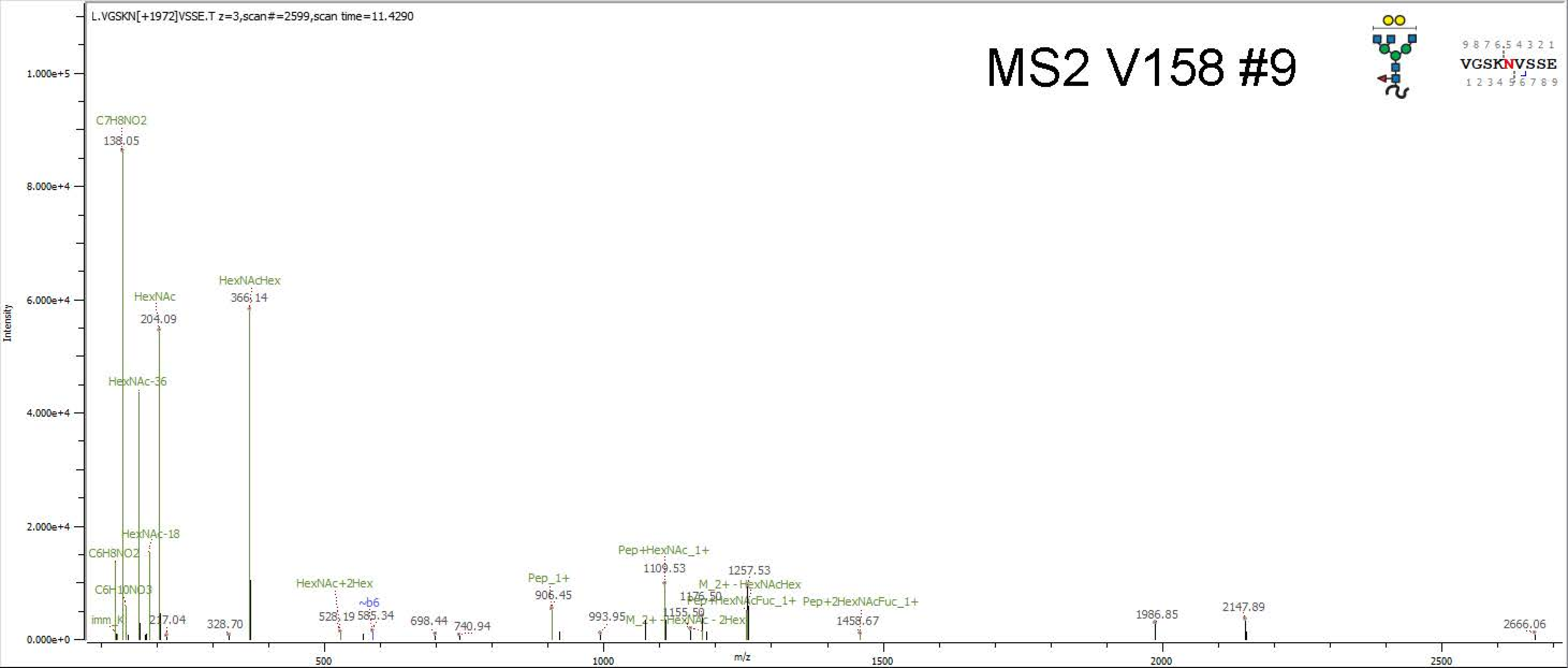
L.VGSKN[+1972]VSSE.T z=3,scan#=2599,scan time=11.4290
MS2 V158 #9
9 8 7 6 5 4 3 2 1
VGSKNVSSE
1 2 3 4 5 6 7 8 9
Intensity
1.000e+5
8.000e+4
6.000e+4
4.000e+4
2.000e+4
0.000e+0
C7H8NO2
138.05
HexNAcHex
366.14
HexNAc
204.09
HexNAc-36
HexNAc-18
C6H8NO2
C6H10NO3
imm_K
217.04
328.70
HexNAc+2Hex
528.19
~b6
585.34
698.44
740.94
Pep_1+
906.45
993.95
M_2+ - HexNAc - 2Hex
Pep+HexNAc_1+
1109.53
1155.50
1176.50
Pep+HexNAcFuc_1+
1257.53
M_2+ - HexNAcHex
Pep+2HexNAcFuc_1+
1458.67
1986.85
2147.89
2666.06
500
1000
m/z
1500
2000
2500

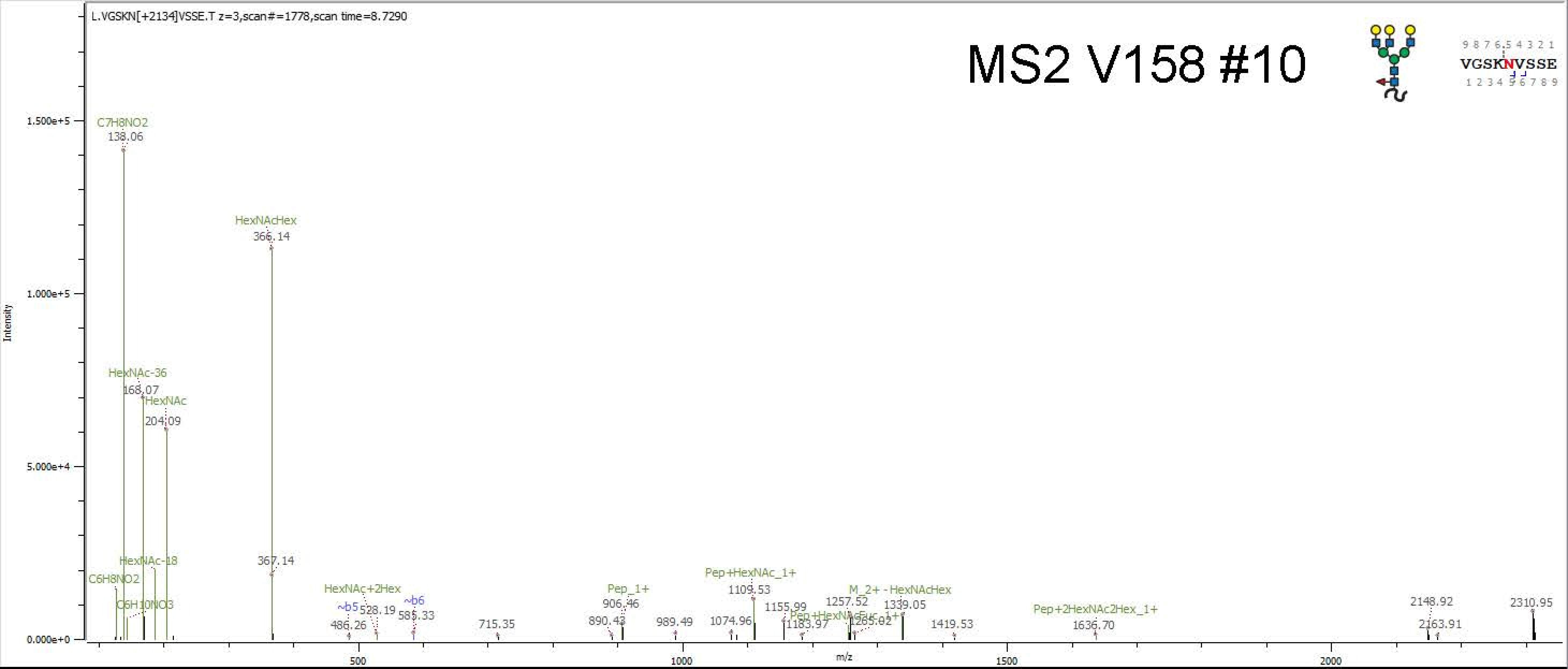

L.VGSKN[+2134]VSSE.T z=3,scan#=1778,scan time=8.7290
MS2 V158 #10
9 8 7 6 5 4 3 2 1
VGSKNVSSE
1 2 3 4 5 6 7 8 9
Intensity
1.500e+5
1.000e+5
5.000e+4
0.000e+0
C7H8NO2
138.06
HexNAcHex
366.14
HexNAc-36
168.07
HexNAc
204.09
HexNAc-18
C6H8NO2
C6H10NO3
367.14
HexNAc+2Hex
~b5
528.19
486.26
~b6
585.33
715.35
Pep_1+
906.46
890.43
989.49
Pep+HexNAc_1+
1109.53
1074.96
1155.99
1183.97
1257.52
M_2+ - HexNAcHex
1339.05
1265.02
1419.53
Pep+2HexNAc2Hex_1+
1636.70
2148.92
2163.91
2310.95
500
1000
1500
2000
m/z

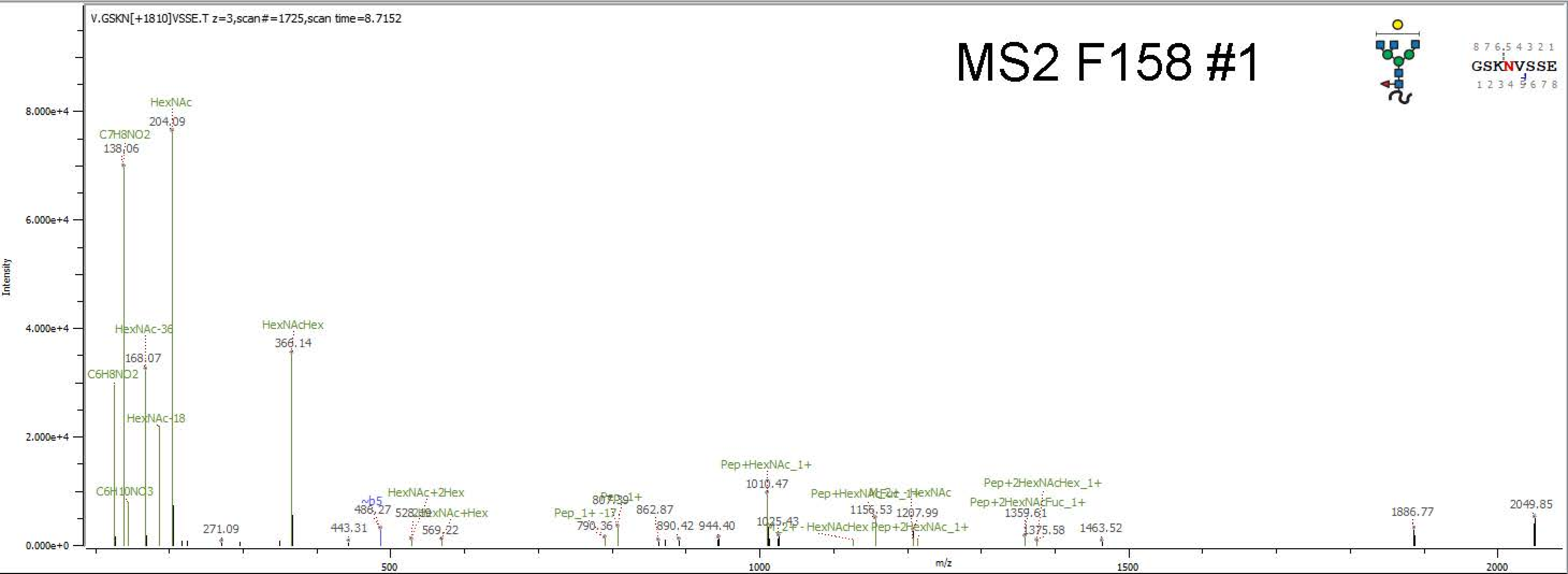

V.GSKN[+1810]VSSE.T z=3,scan#=1725,scan time=8.7152
MS2 F158 #1
GSKNVSSE
HexNAc
204.09
C7H8NO2
138.06
HexNAc-36
168.07
C6H8NO2
HexNAc-18
C6H10NO3
HexNAcHex
366.14
271.09
443.31
486.27
HexNAc+2Hex
569.22
790.36
862.87
890.42
944.40
Pep+HexNAc_1+
1010.47
1025.43
1156.53
1207.99
Pep+2HexNAc_1+
Pep+2HexNAcHex_1+
Pep+2HexNAcFuc_1+
1359.61
1375.58
1463.52
1886.77
2049.85
Intensity
m/z

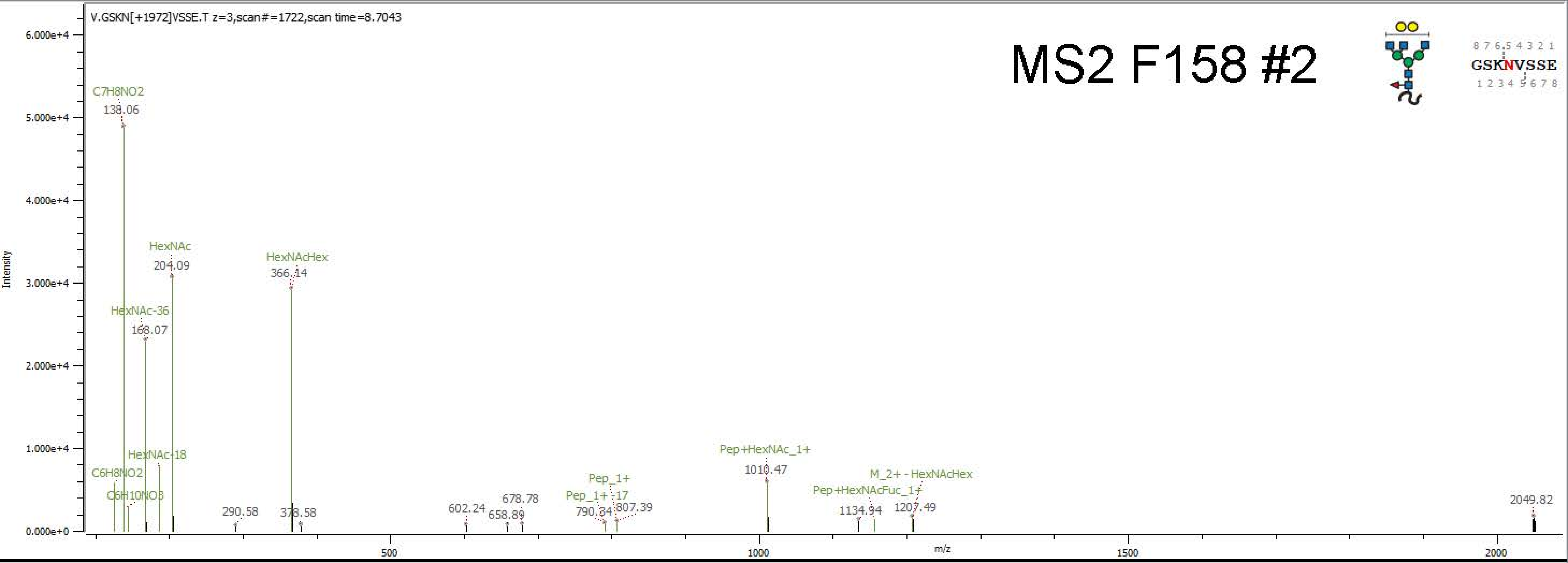

V.GSKN[+1972]VSSE.T z=3,scan#=1722,scan time=8.7043
MS2 F158 #2
8 7 6 5 4 3 2 1
GSKNVSSE
1 2 3 4 5 6 7 8
C7H8NO2
138.06
HexNAc
204.09
HexNAcHex
366.14
HexNAc-36
168.07
HexNAc-18
C6H8NO2
C6H10NO3
290.58
378.58
602.24
658.89
678.78
Pep_1+
Pep_1+ -17
790.34
807.39
Pep+HexNAc_1+
1010.47
Pep+HexNAcFuc_1+
1134.94
M_2+ - HexNAcHex
1207.49
2049.82
Intensity
6.000e+4
5.000e+4
4.000e+4
3.000e+4
2.000e+4
1.000e+4
0.000e+0
500
1000
1500
2000
m/z

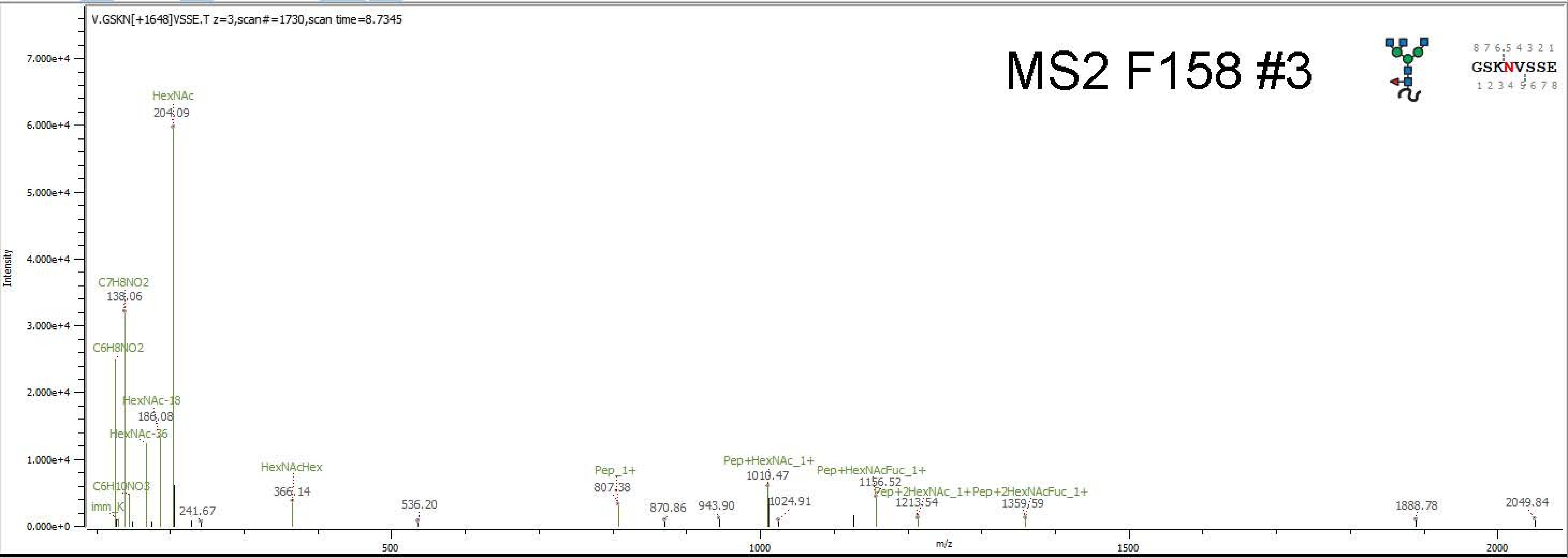

V.GSKN[+1648]VSSE.T z=3,scan#=1730,scan time=8.7345
MS2 F158 #3
8 7 6 5 4 3 2 1
GSKNVSSE
1 2 3 4 5 6 7 8
Intensity
7.000e+4
6.000e+4
5.000e+4
4.000e+4
3.000e+4
2.000e+4
1.000e+4
0.000e+0
HexNAc
204.09
C7H8NO2
138.06
C6H8NO2
HexNAc-18
186.08
HexNAc-36
C6H10NO3
imm_K
241.67
HexNAcHex
366.14
536.20
Pep_1+
807.38
870.86
943.90
Pep+HexNAc_1+
1010.47
1024.91
Pep+HexNAcFuc_1+
1156.52
Pep+2HexNAc_1+
1213.54
Pep+2HexNAcFuc_1+
1359.59
1888.78
2049.84
500
1000
1500
2000
m/z
